# Decoy-resistant IL-18 reshapes the tumor microenvironment and enhances rejection by anti–CTLA-4 in renal cell carcinoma

**DOI:** 10.1172/jci.insight.184545

**Published:** 2024-11-19

**Authors:** David A. Schoenfeld, Dijana Djureinovic, David G. Su, Lin Zhang, Benjamin Y. Lu, Larisa Kamga, Jacqueline E. Mann, John D. Huck, Michael Hurwitz, David A. Braun, Lucia Jilaveanu, Aaron M. Ring, Harriet M. Kluger

**Affiliations:** 1 Section of Medical Oncology and; 2 Section of Surgery, Yale School of Medicine, New Haven, Connecticut, USA.; 3 Department of Internal Medicine, Emory University School of Medicine, Atlanta, Georgia, USA.; 4 Department of Immunobiology, Yale School of Medicine, New Haven, Connecticut, USA.; 5 Immunotherapy Integrated Research Center, Fred Hutchinson Cancer Center, Seattle, Washington, USA.

**Keywords:** Immunology, Oncology, Cancer, Cytokines, Immunotherapy

## Abstract

The cytokine IL-18 has immunostimulatory effects but is negatively regulated by a secreted binding protein, IL-18BP, that limits IL-18’s anticancer efficacy. A decoy-resistant form of IL-18 (DR-18) that avoids sequestration by IL-18BP while maintaining its immunostimulatory potential has recently been developed. Here, we investigated the therapeutic potential of DR-18 in renal cell carcinoma (RCC). Using pantumor transcriptomic data, we found that clear cell RCC had among the highest expression of IL-18 receptor subunits and *IL18BP* of tumor types in the database. In samples from patients with RCC treated with immune checkpoint inhibitors, IL-18BP protein expression increased in the tumor microenvironment and in circulation within plasma in nonresponding patients, and it decreased in the majority of responding patients. We used immunocompetent RCC murine models to assess the efficacy of DR-18 in combination with single- and dual-agent anti–PD-1 and anti–CTLA-4. In contrast to preclinical models of other tumor types, in RCC models, DR-18 enhanced the activity of anti–CTLA-4 but not anti–PD-1 treatment. This activity correlated with intratumoral enrichment and clonal expansion of effector CD8^+^ T cells, decreased Treg levels, and enrichment of proinflammatory antitumor myeloid cell populations. Our findings support further clinical investigation of the combination of DR-18 and anti–CTLA-4 in RCC.

## Abstract 2

IL-18 engineered to avoid decoy-receptor binding enhances tumor rejection by anti-CTLA-4 in kidney cancer models through immune microenvironment remodeling.

## Introduction

In recent years, the treatment paradigm for advanced renal cell carcinoma (aRCC) has shifted, with the emergence of immune checkpoint inhibitors (ICIs) that target CTLA-4 and PD-1 and newer-generation vascular endothelial growth factor receptor–targeting (VEGFR-targeting) tyrosine kinase inhibitors (TKIs). Combination regimens of dual ICIs targeting CTLA-4 and PD-1, or anti–PD-1 plus a TKI, have significantly extended overall survival compared with previous therapies (1–4). Still, many patients do not respond to front-line therapy, and among initial responders, responses are usually transient (5). There is substantial need for novel therapeutic approaches in RCC beyond traditional ICIs. Given the demonstrated immune responsiveness of RCC, new immunomodulatory agents represent a promising area for investigation (6).

Cytokine-based therapies represent one such approach. High-dose IL-2 and IFN-α have been used for decades in aRCC, albeit with low response rates (7). Other cytokine-based therapies, including IL-12, IL-15, and IL-21, are being explored (7). IL-18 is another potential anticancer cytokine. A member of the IL-1 cytokine family, IL-18 can stimulate innate lymphocytes and activate antigen-experienced T cells, and it is a potent inducer of IFN-γ (8). Due to its immunostimulatory effects, recombinant IL-18 was previously tested in early-phase clinical trials, and while it was safe and well tolerated, it lacked efficacy in melanoma (9, 10). However, IL-18 is negatively regulated by a secreted protein (IL-18 binding protein [IL-18BP]) that binds to IL-18 with high affinity and thus prevents its interaction with the IL-18 receptor (11). Levels of IL-18BP increased in response to administration of recombinant IL-18, suggesting that IL-18BP may have abrogated maximal activity of IL-18 therapy (9).

A recent study demonstrated that IL-18BP is highly expressed in various cancers, including clear cell RCC (ccRCC) and that it functions as a secreted immune checkpoint in cancer (12). Directed evolution was used to engineer a modified version of IL-18, termed “decoy-resistant” IL-18 (DR-18), which avoids neutralization by IL-18BP while maintaining its immune cell–stimulating potential. DR-18 exerted potent antitumor effects in mouse models of melanoma and colon cancer by remodeling the immune tumor microenvironment (TME) and activating antigen-specific CD8^+^ tumor-infiltrating lymphocytes (TILs), which were sufficient to induce antitumor responses. Anti–PD-1 enhanced the activity of DR-18 in the initial models tested. DR-18 also inhibited tumor growth in MHC class I–deficient tumors, a major mechanism of ICI resistance, through NK cell activity. DR-18 thus represents a promising therapeutic agent with the potential to synergize with ICIs and have activity in ICI-resistant settings. Accordingly, the first-in-human trial of the human version of DR-18 is currently underway to evaluate safety, pharmacokinetics, pharmacodynamics, and clinical activity in patients with relapsed or refractory solid tumors (NCT04787042; https://clinicaltrials.gov/study/NCT04787042).

Based on these preclinical data, the particularly high expression of *IL18BP* in ccRCC (12), and the demonstrated responsiveness of RCC to ICIs and other cytokine-based immunotherapies, we hypothesized that IL-18 could be an effective cytokine for treating aRCC. Herein, we investigated IL-18BP and the IL-18 receptor in samples from patients with RCC and determined the antitumor activity of DR-18 in RCC murine models and the combined effects with different ICIs.

## Results

### ccRCC has high expression of IL-18 receptor subunits (IL18R1 and IL18RAP) and IL18BP.

**Figure 1:**
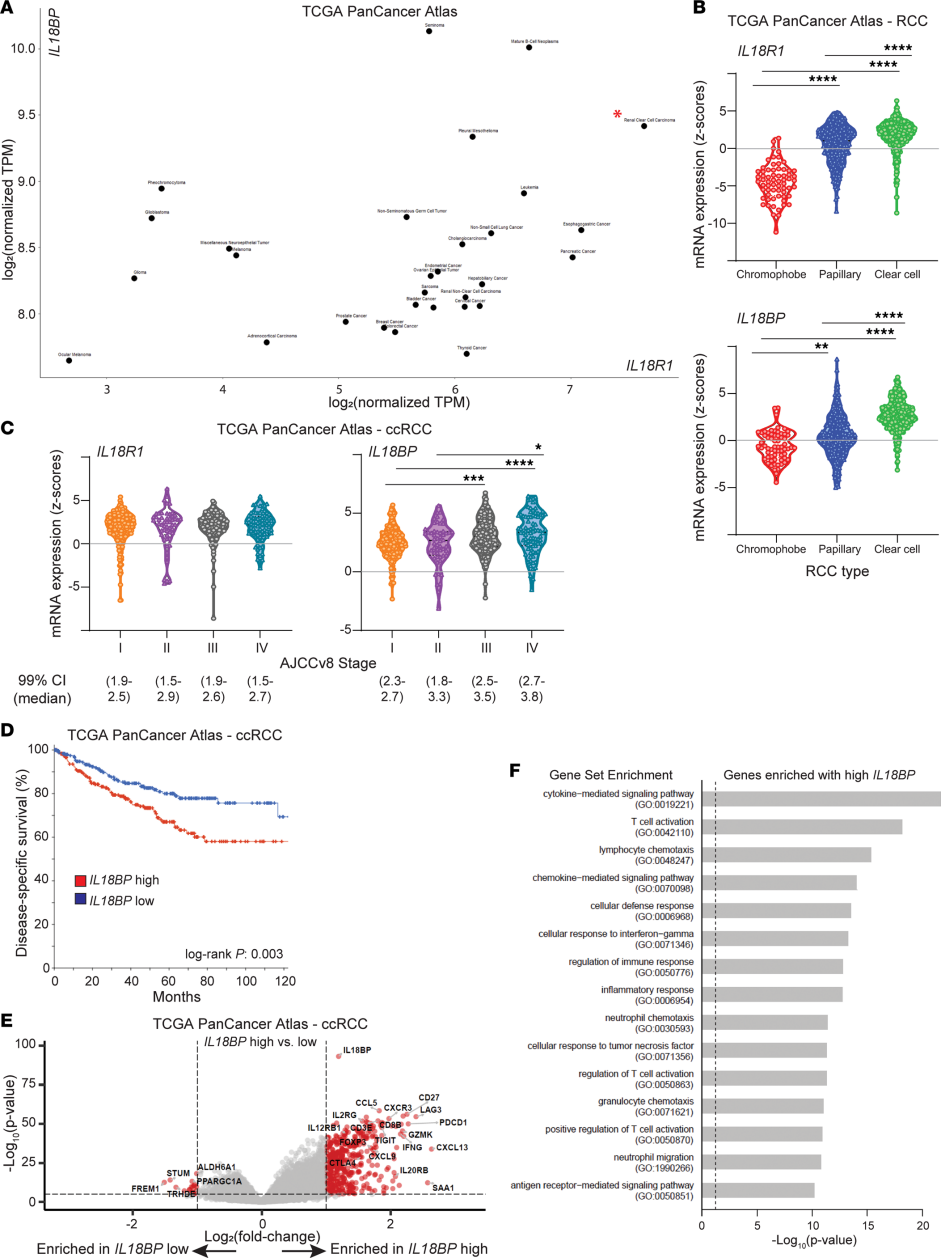
*IL18BP* and *IL18R1* are expressed at high levels in ccRCC and elevated *IL18BP* is associated with cytokine and T cell activation and worse survival. (**A**–**C**) *IL18R1* and *IL18BP* expression from TCGA PanCancer Atlas for all tumors (ccRCC indicated with red asterisk) (**A**), RCC histologic subtypes (**B**), and for ccRCC (**C**), by stage. (**D**) Kaplan-Meier survival curves based on *IL18BP* expression in ccRCC, dichotomized by median expression. (**E**) Volcano plot of transcripts enriched with high versus low *IL18BP* expression in ccRCC (log_2_ fold-change thresholds of 1 and –1; *P* value threshold of 1 × 10^–6^). (**F**) The top gene sets from enrichment analysis of transcripts enriched with high *IL18BP* expression. For **B** and **C**, statistical testing was performed using Kruskal-Wallis test with Dunn’s correction for multiple comparisons. **P < 0.01; ***P < 0.001; ****P < 0.0001.

We employed The Cancer Genome Atlas (TCGA) PanCancer data to determine mRNA expression of IL-18 receptor subunits (*IL18R1* and *IL18RAP*) and *IL18BP* in RCC. ccRCC has among the highest expression of both IL-18 receptor subunits and *IL18BP* relative to 29 other cancer types (Figure 1A and Supplemental Figure 1A; supplemental material available online with this article; https://doi.org/10.1172/jci.insight.184545DS1). Comparing across the 3 most common RCC histologic subtypes (clear cell, papillary, and chromophobe), expression of IL-18 receptor subunits and *IL18BP* was highest in ccRCC and lowest in chromophobe RCC (Figure 1B and Supplemental Figure 1B). Higher *IL18BP* but not IL-18 receptor subunit expression was associated with higher disease stage in ccRCC (Figure 1C and Supplemental Figure 1C). Higher *IL18BP* expression in ccRCC was also associated with higher tumor grade, higher hypoxia signatures scores, and worse survival (Figure 1D and Supplemental Figure 1, D and E). Transcriptional analysis revealed that ccRCC tumors with high *IL18BP* expression are enriched for markers of cytokine/chemokine signaling, T cell activation, and neutrophil/granulocyte chemotaxis (Figure 1, E and F, Supplemental Figure 2A, Supplemental Table 1). Numerous immune checkpoints were among the most significantly upregulated genes with high *IL18BP* expression, and *IL18BP* expression was highly correlated with *LAG3*, *TIGIT*, *PDCD1*, and *CTLA4* expression, as well as expression of *CD4* and the Treg marker *FOXP3* (Supplemental Figure 2B and Supplemental Table 2). *IL18BP* and *IL18* levels were also significantly correlated, although to a lesser degree (Supplemental Figure 2C). Altogether, these findings suggest that the IL-18/IL-18BP axis may play an important role in shaping the TME in at least a subset of ccRCC tumors.

### IL-18BP protein expression increases after ICIs in nonresponding patients with RCC.

**Figure 2:**
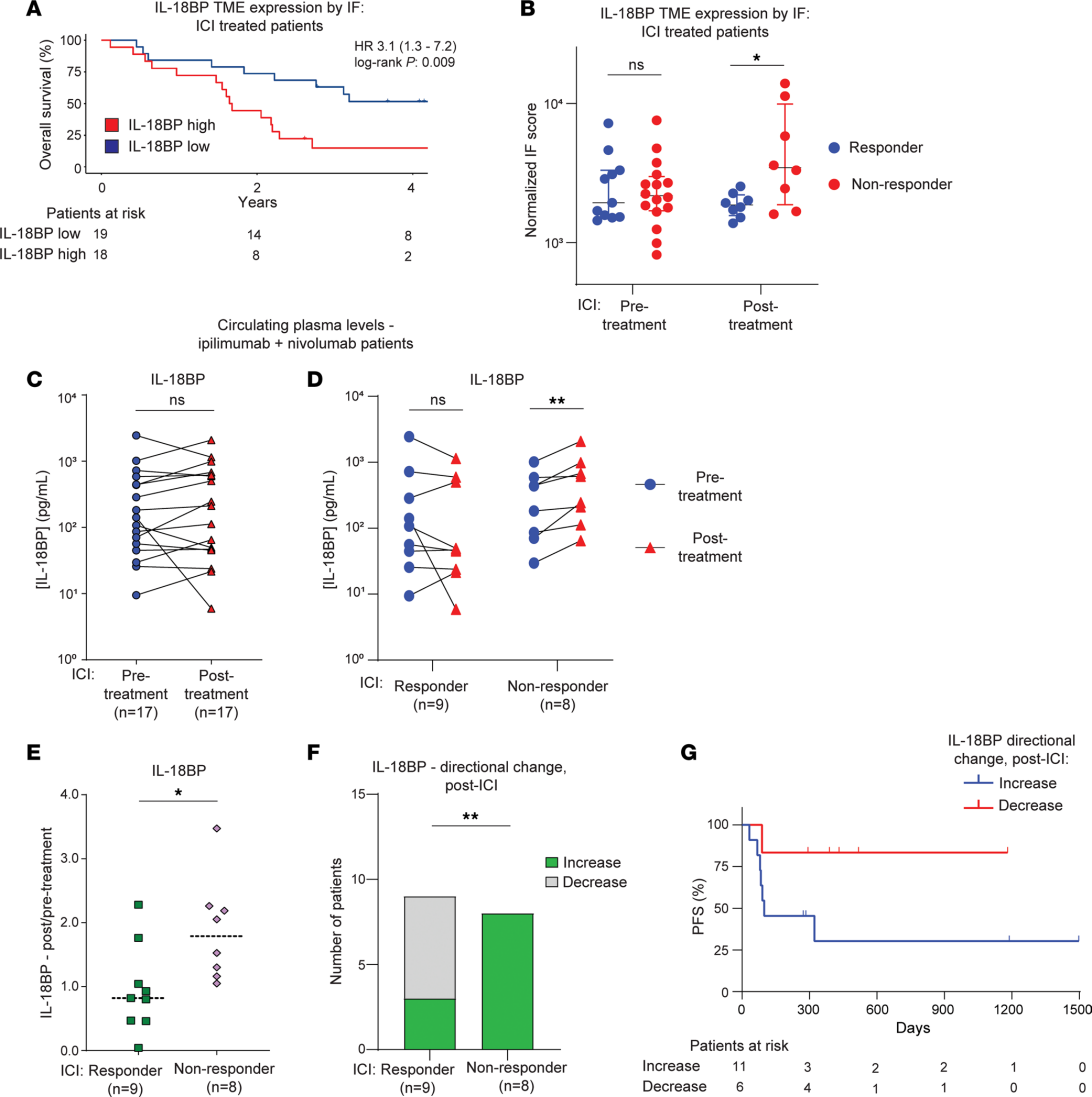
IL-18BP protein levels increase after immunotherapy in nonresponding patients with RCC. (**A**) Kaplan-Meier curves of overall survival of patients with RCC after ICIs by IL-18BP protein expression, dichotomized by median qIF levels. (**B**) IL-18BP protein levels assessed by qIF in the same RCC patient cohort as **A**, before and after ICIs, in ICI responders/nonresponders. (**C**) Circulating plasma levels of IL-18BP, as assessed by ELISA, from patient-matched samples before and after ipi + nivo treatment in a different RCC patient cohort from **A** and **B**. (**D**) Circulating plasma levels of IL-18BP from patient-matched samples before- and during ipi + nivo treatment, separated by treatment response. (**E**) The ratio of post/pretreatment IL-18BP plasma levels by treatment response. (**F**) The directional change of IL-18BP plasma levels after treatment by response. (**G**) Kaplan-Meier curves of progression-free survival (PFS) after ipi + nivo by directional change in circulating IL-18BP levels after treatment, in the same RCC cohort as in **C**–**F**. Statistical testing was performed using Mann-Whitney *U* test (**B** and **E**), Wilcoxon matched-pairs signed rank test (**C** and **D**), and Fisher’s exact test (**F**). Due to small samples sizes, formal statistical testing was not conducted on **G**, and the analysis should be viewed as hypothesis generating. *P < 0.05; **P < 0.01.

We next quantified IL-18BP protein expression in the TME using a well-established method of quantitative immunofluorescence (qIF) employing tissue microarrays (TMAs) of human RCC samples (Supplemental Table 3) (13, 14). Representative histospot staining patterns are shown in Supplemental Figure 3A. IL-18BP was expressed in both primary RCC tumors and metastases, with lower expression in brain metastases (Supplemental Figure 3B). Among patients treated with ICI-based therapies (treatment regimens shown in Supplemental Table 3), higher IL-18BP expression was associated with worse overall survival (Figure 2A). IL-18BP levels also significantly increased after immunotherapy in nonresponding patients (stable or progressive disease) (Figure 2B).

To determine if these findings extended beyond the TME, we quantified circulating plasma levels of IL-18BP using ELISA in patients with RCC before and after treatment with ipilimumab and nivolumab (ipi + nivo) in the frontline setting (Supplemental Table 4). Patient-matched plasma IL-18BP levels did not significantly change with treatment (Figure 2C). However, when the patients were separated by response to ipi + nivo, a treatment effect was apparent: in responders (complete or partial responses), plasma IL-18BP levels did not significantly change with ipi + nivo, but they increased significantly in nonresponders, consistent with the qIF data (Figure 2, D and E, and Supplemental Figure 3C). Notably, while plasma IL-18BP levels increased in 100% of nonresponders after treatment, they decreased in 67% of responders (Figure 2F). Furthermore, we found that patients whose plasma IL-18BP levels decreased after immunotherapy had longer progression-free survival (Figure 2G). We did not observe the same patterns with circulating plasma levels of IL-18. Patient-matched plasma IL-18 levels increased after treatment with ipi + nivo but did so at equivalent levels between responders and nonresponders (Supplemental Figure 3, D–G). No differences in circulating IL-18 levels were observed between responders and nonresponders before or after treatment, with nearly all patients having increased plasma IL-18 levels after treatment (Supplemental Figure 3H). Interestingly, circulating IL-18 and IL-18BP levels were significantly correlated in responders, particularly before treatment, while levels were not correlated in nonresponders (before or after treatment) (Supplemental Figure 3I).

### DR-18 in combination with anti–CTLA-4 demonstrates enhanced in vivo activity in RCC and melanoma murine models.

**Figure 3:**
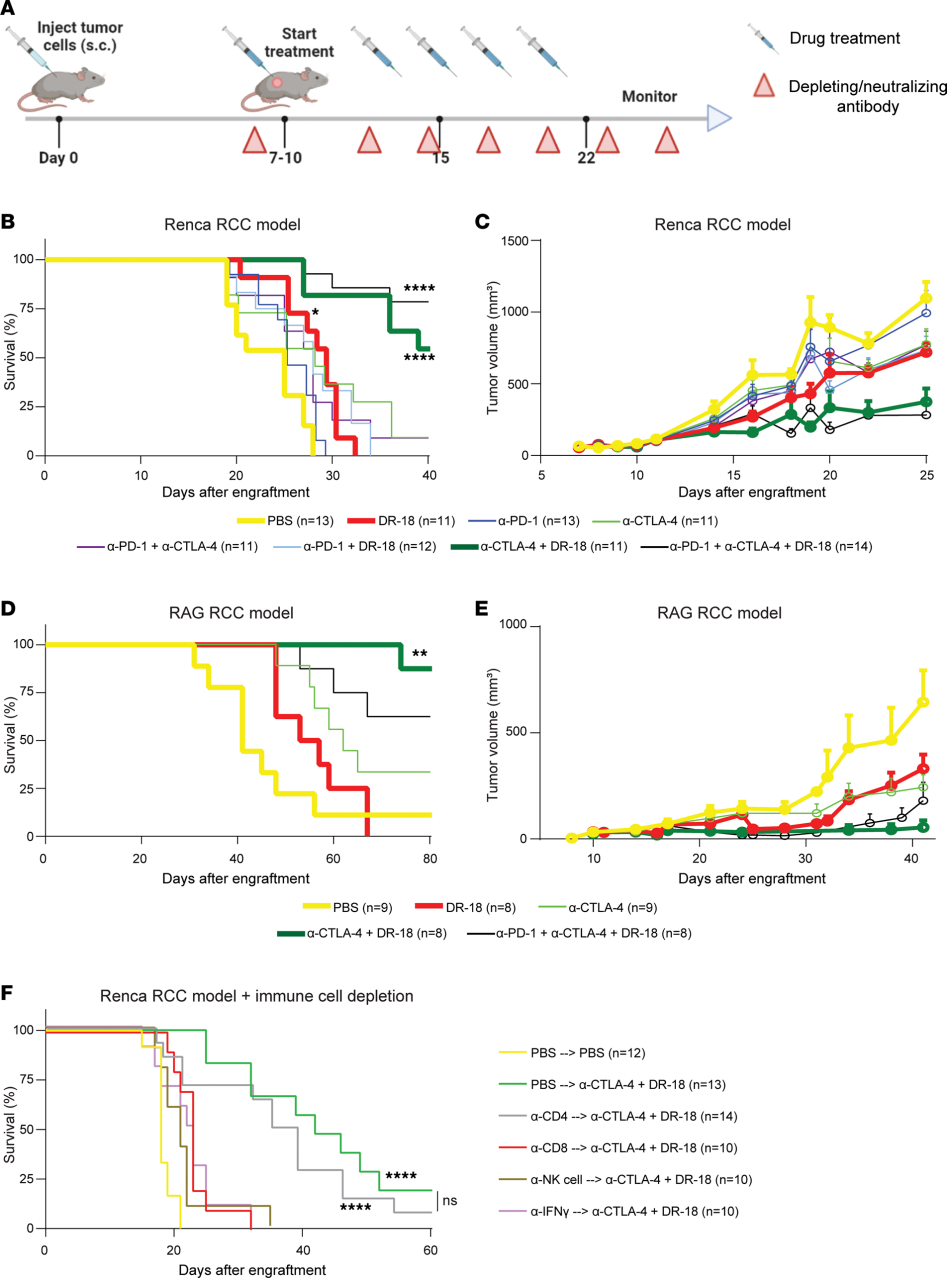
DR-18 combined with anti–CTLA-4 extends survival in murine RCC models. (**A**) WT immunocompetent balb/c mice were s.c. engrafted with 0.5 × 10^6^ Renca or 1.0 × 10^6^ RAG cells. Starting on day 7–10, mice were treated twice weekly with phosphate buffered saline (PBS), DR-18 (s.c.), and/or ICIs (anti–PD-1/anti–CTLA-4) i.p. Five treatments were given. Red triangles indicate timing of administration of depleting/neutralizing antibodies. (**B**–**E**) Kaplan-Meier survival curves and mean tumor growth curves of mice engrafted with Renca (**B** and **C**) and RAG (select treatment groups shown) (**D** and **E**) cells. Data are shown as mean ± SEM (**C** and **E**). (**F**) Survival of mice engrafted with Renca tumors and treated with control PBS or DR-18 + anti–CTLA-4, either alone (PBS depletion) or with depleting/neutralizing antibodies. Depleting/neutralizing antibodies were given 24 hours prior to treatment and twice weekly thereafter. NK cells were depleted using anti-Asialo GM1. Renca data were combined from 3 independent experiments; RAG data were combined from 2 independent experiments. For Kaplan-Meier curves, statistical testing was performed using the log-rank test with Bonferroni correction in comparison with control-treated mice. *P < 0.05; **P < 0.01; ****P < 0.0001

Having seen that the IL-18 pathway may be primed for reactivation in ccRCC, we next performed tumor growth and survival analyses in 2 syngeneic, immunocompetent murine RCC models: Renca and RAG (15, 16). We tested DR-18 monotherapy and combination therapy with single- and dual-agent ICIs, including both anti–PD-1 and anti–CTLA-4 targeting antibodies (Figure 3A). In the Renca model, DR-18 monotherapy modestly inhibited tumor growth and prolonged survival, comparable with ICIs (Figure 3, B and C, and Supplemental Figure 4A). Interestingly, adding PD-1 blockade to DR-18 did not enhance efficacy, whereas the addition of anti–CTLA-4 to DR-18 significantly increased antitumor effects. Triple-therapy (DR-18 + anti–PD-1 + anti–CTLA-4) did not further inhibit tumor growth or prolong survival compared with the doublet (DR-18 + anti–CTLA-4). The RAG model was more sensitive to ICIs but produced similar results, again showing a greater effect of anti–CTLA-4 than anti–PD-1 when combined with DR-18 (Figure 3, D and E, and Supplemental Figure 4, B–D). Immune cell depletion studies in the Renca model demonstrated that CD8^+^ T cells, NK cells, and IFN-γ, but not CD4^+^ T cells, are similarly required for activity of DR-18 + anti–CTLA-4 (Figure 3F). We conclude that DR-18 monotherapy has modest activity in murine RCC models, but the combination of DR-18 + anti–CTLA-4 may be particularly effective.

We then investigated whether the efficacy of DR-18 + anti–CTLA-4 extended beyond RCC models. In the murine melanoma model YUMMER1.7, DR-18 was efficacious as a monotherapy and demonstrated added activity with anti–PD-1 (Supplemental Figure 4, E and F) (12). DR-18 + anti–PD-1 efficacy was comparable with dual-agent ICIs in YUMMER1.7 and was higher than in the RCC models. DR-18 + anti–CTLA-4 was equally as effective as these regimens in the YUMMER1.7 model.

In the RAG and YUMMER1.7 models, where multiple mice treated with various drug regimens had complete tumor regression and prolonged responses, tumor rechallenge studies with twice the initial dose of tumor cells were performed. In all mice tested, no tumors grew out on rechallenge regardless of the initial treatment regimen, indicating prolonged antitumor memory responses.

### DR-18 in combination with anti–CTLA-4 induces a broad inflammatory response.

**Figure 4:**
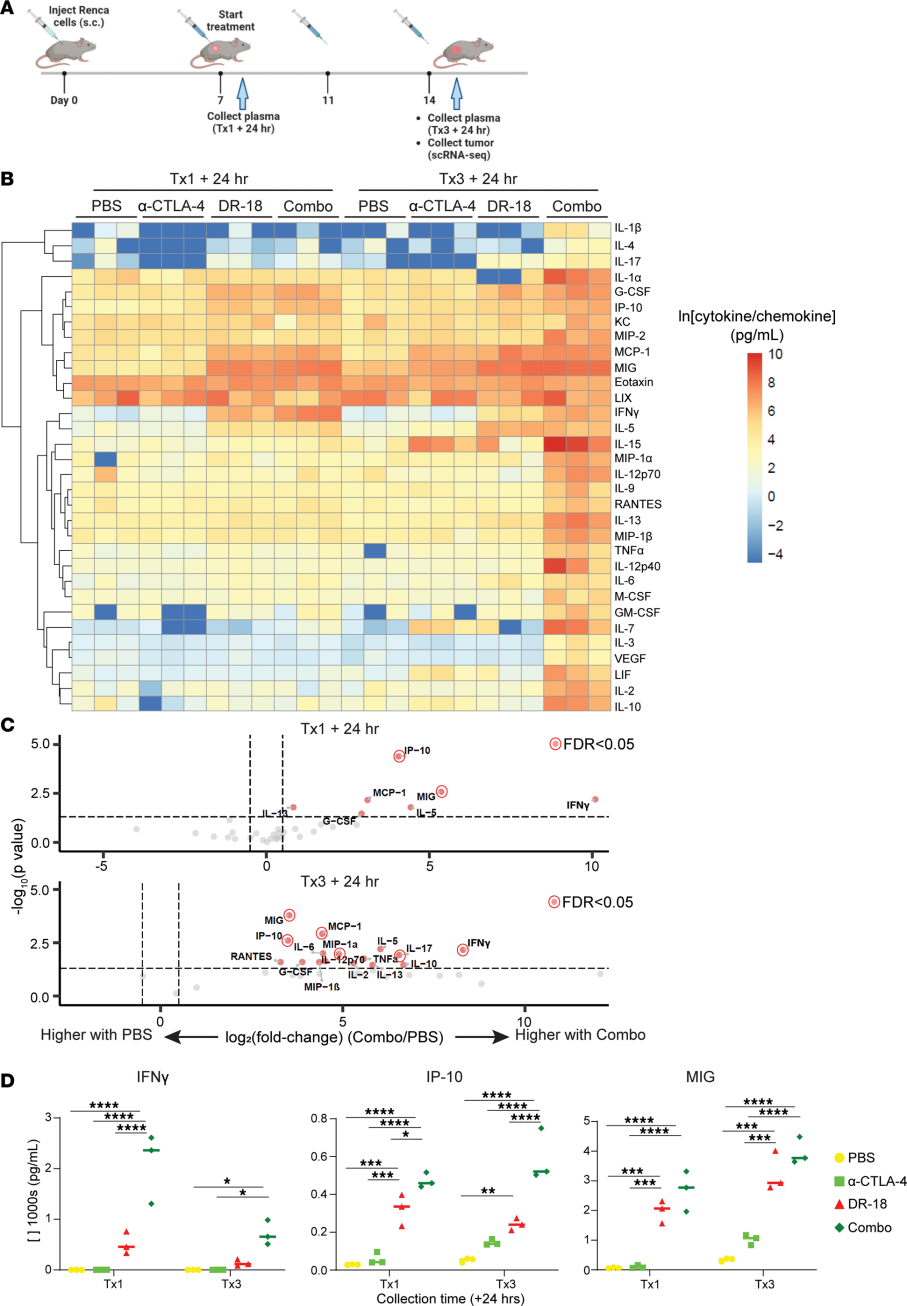
DR-18 + anti–CTLA-4 potently induces inflammatory cytokines/chemokines. (**A**) Schematic of treatment and sample collection time points for cytokine/chemokine profiling and scRNA/TCR-Seq in the Renca model. (**B**) Heatmap of the natural logarithm of circulating cytokine/chemokine levels in mice for the indicated treatments and time points (*n* = 3 mice/group, with the same mice collected at each time point), with unsupervised hierarchical clustering on the *y* axis. Data were generated using 31-plex Mouse Cytokine/Chemokine Array from Eve Technologies (MD31). (**C**) Volcano plots of the same data as in **B**, comparing circulating cytokine/chemokine levels with DR-18 + anti–CTLA-4 treatment (Combo) to PBS (log_2_ fold-change thresholds of 0.5 and –0.5; *P* value threshold of 0.05; cytokine/chemokine changes with FDR < 0.05 highlighted as indicated). (**D**) Absolute levels of the indicated cytokines/chemokines at each time point for each treatment. Statistical testing performed using 2-way ANOVA with Tukey’s multiple comparisons test comparing all conditions within a given time point; only significant comparisons are shown. Tx, treatment; hr, hours. *P < 0.05; ***P < 0.001; ****P < 0.0001

We then sought to understand how the combination of DR-18 and anti–CTLA-4 alters the mouse immune system. To start, we profiled circulating cytokines/chemokines in mice with Renca tumors after 2 different time points of treatment with single-agent or combination DR-18 + anti–CTLA-4 (Figure 4A). After the first treatment, DR-18–containing regimens produced increases in multiple inflammatory cytokines, including IFN-γ, IP-10 (CXCL10), MIG (CXCL9), IL-5, G-CSF, and MCP-1 (CCL2) (Figure 4, B–D). Increases in IFN-γ, IP-10, and MIG were particularly pronounced with DR-18 + anti–CTLA-4 treatment (Figure 4, C and D). Of note, IP-10 and MIG are known to be induced by IFN-γ. After the third treatment, these and most of the other cytokines/chemokines profiled were elevated in the DR-18 + anti–CTLA-4–treated mice, suggesting the induction of a broad inflammatory response by this point in the treatment course, including Th1, Th2, and Th17 programs.

### Enrichment and clonal expansion of effector CD8^+^ T cells with DR-18 + anti–CTLA-4.

**Figure 5:**
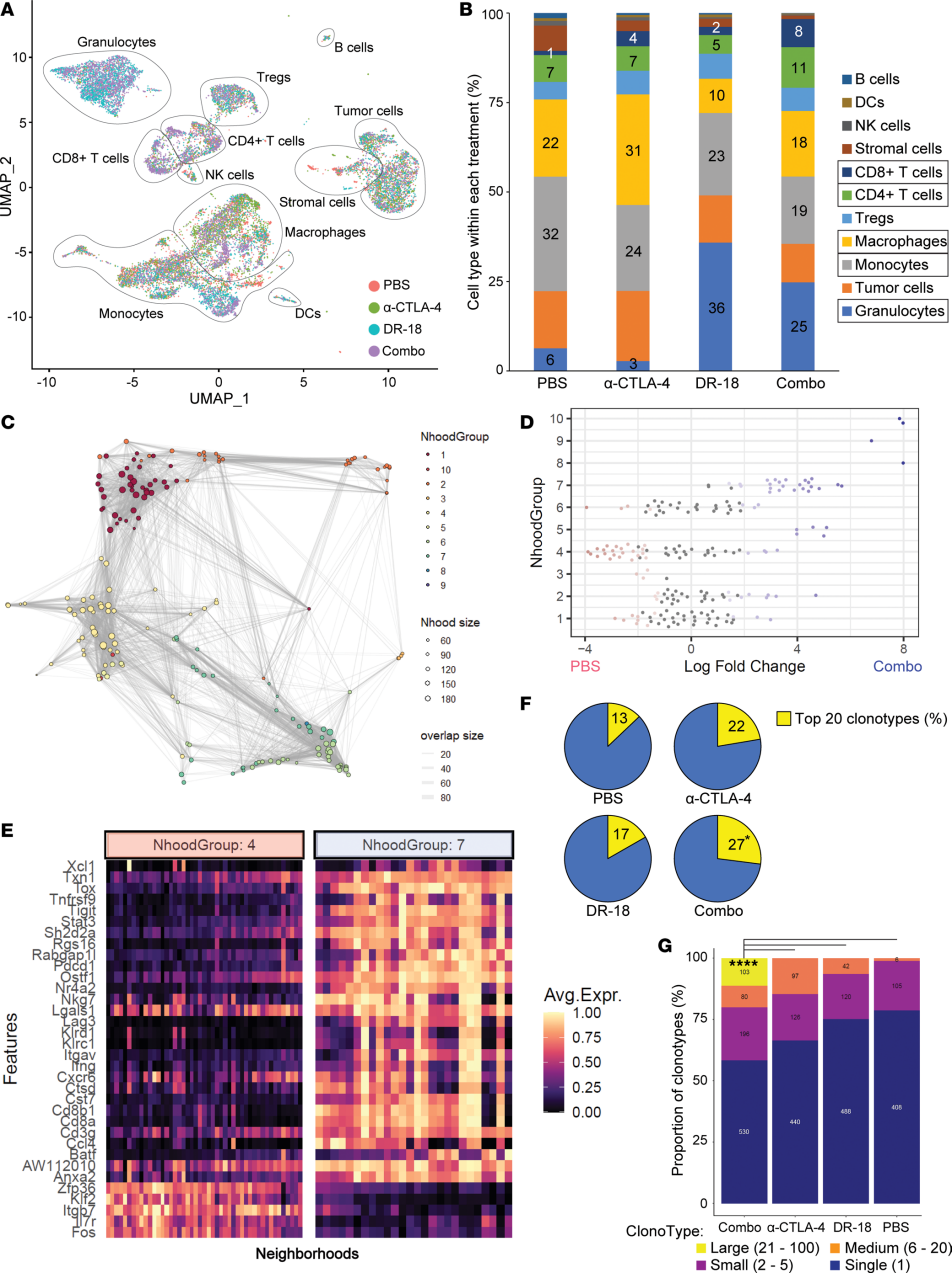
DR-18 alters immune subset composition in Renca tumors, including enrichment and clonal expansion of CD8^+^ effector T cells. (**A**) Uniform Manifold Approximation and Projection (UMAP) dimensionality reduction plot of clustering and annotation of all cell populations isolated from Renca tumors treated for 3 cycles with PBS, DR-18, anti–CTLA-4, or DR-18 + anti–CTLA-4 (Combo) (*n* = 3 mice/group, pooled) based on scRNA-Seq analysis. Annotations were performed using SingleR. (**B**) Quantification of the proportion of each cell population from **A** within each of the treatment groups, showing enrichment of granulocytes with DR-18 treatment and CD8^+^ and CD4^+^ T cells with DR-18 + anti–CTLA-4. For select cell populations (boxed), the percentages within each treatment group are shown. (**C**) Neighborhood group plot from Milo analysis of T cell subsets from scRNA-Seq data. (**D**) Differential abundance fold changes of the neighborhood groups in **C**, comparing the Combo treatment with control, showing enrichment and deenrichment of certain groups. (**E**) Heatmap of the top differentially expressed genes between neighborhood group #7, enriched with DR-18 + anti–CTLA-4 treatment and with high expression levels of markers of T cell activation, cytolytic activity, and exhaustion, versus neighborhood group #4, deenriched with combination treatment. (**F**) Relative proportion of the top 20 clonotypes out of the total for each treatment group based on TCR analysis. (**G**) Clonotype proportions by size category based on TCR analysis, showing clonal expansion with DR-18 + anti–CTLA-4 (Combo). Statistical testing performed using Fisher’s exact test comparing control with all other treatment conditions, with only significant comparisons shown **F**, and χ^2^ test comparing DR-18 + anti–CTLA-4 (Combo) to all other conditions. *P < 0.05; ****P < 0.0001

To gain insight into global changes to the TME with DR-18, anti–CTLA-4, or the combination, we performed single-cell RNA and T cell receptor (TCR) sequencing (scRNA-Seq and scTCR-Seq) of Renca tumors with and without treatment (Figure 4A). A comparison of the proportion of different infiltrating immune cell types revealed largescale changes in granulocytes and macrophages/monocytes with DR-18 treatment (Figure 5, A and B, and Supplemental Figure 5, A–D), reproducing prior findings (*P* < 0.0001, control vs. each DR-18 containing regimen, Fisher’s exact test) (12). Only the combination of DR-18 and anti–CTLA-4 led to higher relative CD4^+^ and CD8^+^ T cell infiltration compared with every other regimen (*P* < 0.0001, Fisher’s exact tests) (Figure 5B and Supplemental Figure 5D).

To probe tumor-infiltrating T cell population differences based on treatment groups, we performed differential abundance testing on the T cell subsets using Milo, which assigns cells to partially overlapping neighborhoods on a k-nearest neighbor graph and then groups neighborhoods (Figure 5C and Supplemental Figure 6A) (17). Comparing the most prominent enriched neighborhood group containing a substantial number of neighborhoods (group #7) to the most deenriched (group #4) with combination treatment, we observed enrichment of numerous markers of CD8^+^ T cell activation and cytolytic activity, as well as exhaustion markers, including *Cd8a*, *Tox*, *Klrd1*, *Klrc1*, and *Ifng*, and the immune checkpoints *Tigit*, *Pdcd1*, and *Lag3* (Figure 5, D and E, and Supplemental Figure 6B). Similar analyses of other neighborhood groups revealed deenrichment of Tregs (group #1) and mild enrichment of an activated CD4^+^ T cell population (group #5) (Supplemental Figure 6, C–E).

To verify these findings, we performed additional analysis on the T cell subsets. Unsupervised hierarchical clustering revealed an enriched population of activated CD8^+^ T cells with combination treatment (cluster 0) (Supplemental Figure 7, A–C). Semisupervised analysis with well-annotated reference murine TIL markers similarly demonstrated enrichment of effector CD8^+^ T cells (both precursor and terminally exhausted CD8^+^ populations) as well as a concomitant decrease in CD8^+^ and CD4^+^ naive–like populations, with the combination regimen (Supplemental Figure 7, D–G). While treatment with DR-18 monotherapy elicited a relative increase in Tregs, with the combination of DR-18 and anti–CTLA-4, the relative proportion of Tregs remained stable (Supplemental Figure 7, F and G). Focused analysis of immune checkpoint expression on T cells revealed strong induction of *Ctla4* — and, to a lesser extent, *Pdcd1* and *Tigit* — with DR-18 monotherapy, whereas there was stronger induction of *Pdcd1* and *Tigit* relative to *Ctla4* with DR-18 + anti–CTLA-4 (Supplemental Figure 8A).

Single-cell TCR analysis further demonstrated a greater degree of clonal expansion and loss of clonal diversity after treatment with the combination of DR-18 + anti–CTLA-4 relative to either monotherapy (Figure 5, F and G, and Supplemental Figure 8, B and C). While no single clonotype was detected across all 4 treatment groups, a CD8^+^ clonotype from the DR-18 monotherapy arm expanded to become a dominant effector CD8^+^ clonotype in the combination arm (Supplemental Figure 8, D and E).

### Expansion of proinflammatory myeloid populations with DR-18 + anti–CTLA-4.

**Figure 6:**
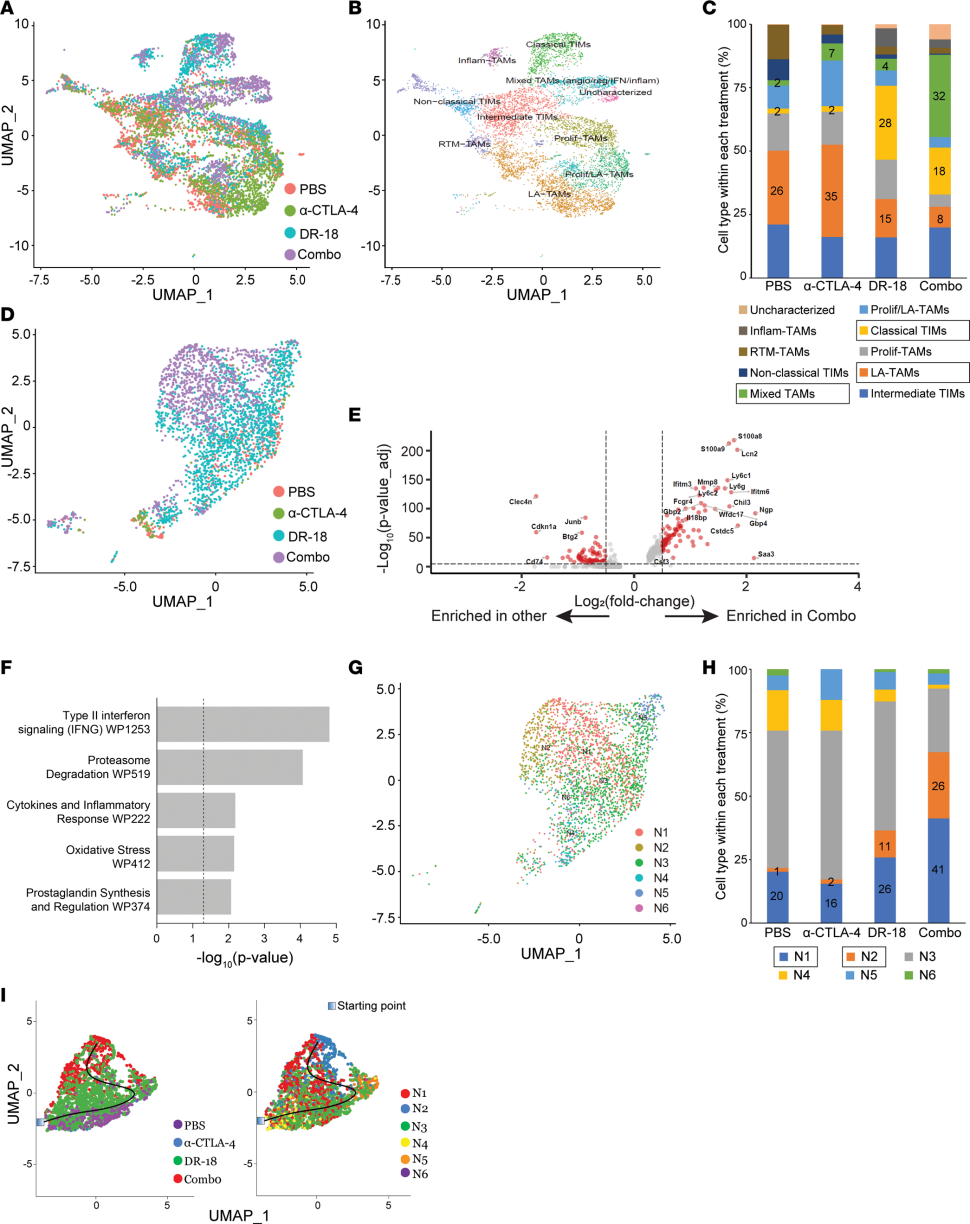
DR-18 + anti–CTLA-4 leads to intratumoral expansion of proinflammatory myeloid populations. (**A** and **B**) UMAP plots of all macrophages/monocytes identified by scRNA-Seq analysis with overlaid treatment groups (**A**) and annotated clusters (**B**). Annotation was performed based on the phenotypic groups and markers described in Ma et al. (18). (**C**) Quantification of the proportion of each macrophage/monocyte subtype from **B** within each of the treatment groups, showing relative enrichment of proinflammatory and loss of protumorigenic subtypes. For select cell populations (boxed), the percentages within each treatment group are shown. (**D**) UMAP plot of all granulocytes identified by scRNA-Seq analysis with overlaid treatment groups. (**E**) Volcano plot of differential gene expression between granulocytes from tumors treated with combination DR-18 + anti–CTLA-4 (Combo) versus all other treatment groups (Other) (log_2_ fold-change thresholds of 0.5 and –0.5; *P* value-adjusted threshold of 1 × 10^–6^). (**F**) The top gene sets from enrichment analysis of genes enriched in granulocytes from Combo-treated tumors. (**G** and **H**) UMAP plot of all neutrophils from scRNA-Seq analysis with overlaid neutrophil subtype classification based on Zilionis et al. (25) (**G**), with quantification of the relative proportion of each subtype by treatment group (**H**). For select cell populations (boxed), the percentages within each treatment group are shown. (**I**) UMAP plots of neutrophils showing trajectory analysis using Slingshot from the given starting point, with overlaid treatment groups (left) and neutrophil subtypes (right), as in **G**.

Given the importance of myeloid populations in immune modulation in RCC, we characterized changes to myeloid populations with DR-18, anti–CTLA-4, and the combination. Unsupervised hierarchal clustering of the monocyte/macrophage subsets suggested population shifts with drug treatment (Figure 6A and Supplemental Figure 9A). To phenotypically classify these clusters, we employed the classification system for murine tumor associated macrophages (TAMs) and tumor-infiltrating monocytes (TIMs) described in Ma et al. (18). This analysis revealed reductions in protumorigenic TAM subtypes, particularly the lipid-associated–TAMs (LA-TAMs), with DR-18 treatment, as well as increased infiltration of classical TIMs, traditionally associated with proinflammatory effects (*P* < 0.0001, control vs. each DR-18 containing regimen, Fisher’s exact test) (Figure 6, B and C, and Supplemental Figure 9, B and C). The DR-18 + anti–CTLA-4 combination also led to the expansion of a TAM population compared with every other regimen (*P* < 0.0001, Fisher’s exact tests) defined by markers from multiple phenotypic subtypes, both proinflammatory and protumorigenic (termed “Mixed TAMs”). This finding aligns with the concept that macrophages exist on a phenotypic and functional spectrum (18–20).

We had also observed increased infiltration of granulocytes after treatment with DR-18, either monotherapy or in combination with anti–CTLA-4, in accord with prior findings (Figure 5B) (12). We hypothesized that phenotypic shifts in granulocyte populations could also be occurring when the combination is given relative to monotherapy, considering the difference in efficacy between the 2 treatments. Unsupervised hierarchal clustering of the granulocyte subsets indeed showed a divergence in granulocyte populations between DR-18 monotherapy and the combination with anti–CTLA-4 (Figure 6D and Supplemental Figure 9D). Differential gene expression analysis revealed enrichment of gene sets associated with type II IFN signaling and cytokines and inflammatory response in granulocytes from combination-treated tumors (Figure 6, E and F).

Recent work has better defined the phenotypic and functional diversity of neutrophils in cancer, which can have both pro- and antitumorigenic roles (21–25). We applied 1 such classification system that has both human and mouse tumor relevance and has been functionally validated in mouse tumor models to our tumor-infiltrating granulocyte population (Figure 6, G and H, and Supplemental Figure 9, E and F) (21, 25). The relative proportions of the N1 and N2 neutrophil subtypes increased with DR-18 + anti–CTLA-4 treatment compared with every other regimen (*P* < 0.0001, Fisher’s exact tests). Of note, these subtypes had been previously identified and functionally validated as playing important roles in tumor control in response to immunotherapy, driven by IFN-γ stimulation downstream of lymphocyte-myeloid cell crosstalk (21, 25). Ligand-receptor network analysis using NicheNet (26) indeed indicated that the neutrophils from DR-18 + anti–CTLA-4–treated tumors were stimulated by IFN-γ produced by CD8^+^ T cells (Supplemental Figure 10A).

We verified the findings from this semisupervised analysis with unsupervised quantitative differential abundance and nearest neighbor clustering analysis using Milo, which showed high levels of enrichment of a neighborhood group (group #4) with combination DR-18 + anti–CTLA-4 treatment that was overlapping with neutrophil subtype N2 and expressed high levels of IFN-response genes (Supplemental Figure 10, B–F). Trajectory analysis showed neutrophils passing through intermediate subtypes before ultimately becoming N1 and then N2 subtypes, coinciding with the pathway to combination DR-18 + anti–CTLA-4 treatment (Figure 6I).

## Discussion

In this study, we investigated the therapeutic potential of DR-18 in RCC. We found that ccRCC tumors express high levels of both IL-18 receptor subunit genes and the secreted blocking protein *IL18BP* relative to other cancer types. Furthermore, an increase in IL-18BP protein expression with ICIs was associated with resistance to treatment in RCC, suggesting that IL-18BP might play a role in poor response to ICIs. Using murine models of RCC, we observed modest antitumor effects from DR-18 monotherapy. However, adding PD-1 blockade to DR-18 did not enhance efficacy, whereas the addition of anti–CTLA-4 to DR-18 significantly increased antitumor effects. This activity correlated with proinflammatory immune microenvironment changes that support therapeutic efficacy.

Our human sample studies implicate circulating IL-18BP and, more specifically, the change in IL-18BP from before to after treatment as a potential predictive biomarker for patients with RCC treated with ICIs. We observed a significant increase in plasma IL-18BP protein levels after initiation of ICIs relative to baseline in nonresponding patients only. Moreover, in a nonoverlapping RCC patient cohort, we observed an increase in tumor IL-18BP protein levels by qIF, indicating that this is both a systemic and local phenomenon. Although our cohort sizes were small, an increase in circulating IL-18BP plasma levels with treatment was found in all 8 nonresponding patients treated with ipi + nivo, while 6 of 9 responding patients had a decrease in circulating IL-18BP. Of note, while these findings need to be verified in larger, independent RCC cohorts, measuring circulating IL-18BP plasma levels at baseline and on treatment (e.g., after 3 cycles of treatment, as done here) would likely not be difficult to implement into clinical practice if indeed the sensitivity and specificity in larger cohorts remain high. It is unclear to what extent these findings extend outside of RCC and ipi + nivo treatment and requires further investigation.

In both syngeneic murine RCC models tested, we found that the combination of DR-18 + anti–CTLA-4 had superior efficacy to either agent alone. This stood in contrast to the combination of DR-18 + anti–PD-1 in these models, which offered little additional benefit relative to each monotherapy. This finding in RCC models differed from the results seen previously in the mouse YUMMER1.7 melanoma model, where DR-18 + anti–PD-1 had increased antitumor effects (12), although in the YUMMER1.7 model enhanced activity was still seen with DR-18 + anti–CTLA-4. These results suggest that the optimal therapy to combine with DR-18 may vary based on tumor type and certain characteristics of the TME.

Single-cell transcriptomic analysis revealed more robust induction of *Ctla4* relative to *Pdcd1* on intratumoral T cells after 3 cycles of treatment with DR-18 monotherapy in the Renca model, offering a potential partial explanation for the superior efficacy of DR-18 + anti–CTLA-4 relative to DR-18 + anti–PD-1 in this model. Additionally, anti–PD-1 and anti–CTLA-4 ICIs are known to have distinct mechanisms of action, with anti–CTLA-4 agents more capable of activating and expanding T cells, particularly CD4^+^ T cells, in the tumor draining lymph nodes, leading to increased trafficking of activated T cells into the TME (27–30). Immune cell depletion experiments in the Renca model, however, showed that partial depletion of CD4^+^ cells with a depleting antibody did not significantly alter the efficacy of DR-18 + anti–CTLA-4, suggesting that CD4^+^ T cells may not be pivotal drivers of antitumor immunity in this particular situation or that smaller numbers of CD4^+^ cells are sufficient to enhance CD8^+^ activity, which appears to be critical. Additionally, the scRNA-Seq T cell subset analysis did not show substantial expansion and activation of effector CD4^+^ populations with DR-18 + anti–CTLA-4 but did indicate deenrichment of Tregs. Anti–CTLA-4 therapy is capable of depleting Tregs in mouse tumor models and some human tumors, and while this is thought to be one of the major mechanisms of anti–CTLA-4 efficacy in mouse models, its role in human tumors is less clear (31–34). The anti–CTLA-4 clone used in this study (9H10) is known to deplete murine Tregs (35). Furthermore, tumors treated with DR-18 monotherapy had increased proportions of Tregs relative to the other treatment groups (nearly twice as many), whereas Treg levels remained stable relative to control-treated tumors with DR-18 + anti–CTLA-4. Altogether, these findings suggest that 1 mechanism of enhanced efficacy of the combination of DR-18 and anti–CTLA-4 is the limitation of DR-18-induced Treg expansion by anti–CTLA-4, although additional studies are needed to unequivocally define the precise mechanisms, including the role of the tumor draining lymph node.

Myeloid populations, including macrophages and neutrophils, are important contributors to antitumor immunity, although they can have pro- and antitumorigenic roles (18). As seen previously (12), we observed shifts in macrophages/monocytes toward more proinflammatory, antitumor phenotypes with DR-18 treatment, changes that were more pronounced when combined with anti–CTLA-4. We also reproduced prior reports showing higher relative neutrophil infiltration with DR-18 (12). The rapid and robust increase in IP-10 and MIG levels with DR-18 treatment implicates these chemokines as possible mediators of this effect, as they are known neutrophil chemoattractants. Persistently high IFN-γ stimulation could also explain the phenotypic shift in neutrophils toward an IFN-stimulated subtype with DR-18 + anti–CTLA-4. The phenotype of these neutrophils was highly overlapping with the N1 and N2 neutrophil subtypes recently identified as vital components of effective antitumor immunity in mouse tumor models (21, 25). Further studies are needed to determine if the neutrophil populations seen in this study play a similar role.

IL-18BP is highly expressed in numerous cancers, including ccRCC (12). While DR-18 has been engineered to avoid sequestration by IL-18BP to enable immune activation, alternative strategies exist to overcome IL-18BP inhibition and could also be investigated in combination with CTLA-4 blockade. Examples of such alternative strategies include use of a decoy-to-the-decoy (36) or monoclonal antibodies targeting IL-18BP (37), both of which would have the effect of increasing endogenous IL-18 activity in the TME. These approaches have the potential of better tolerability, as they would theoretically limit their activity to areas of increased IL-18BP expression, such as the TME. However, as they rely on endogenous IL-18, they may also have lower efficacy and may not be effective for all tumor types or anatomic sites of disease, depending on patterns of IL-18 expression. Additionally, in some situations, IL-18 has demonstrated protumorigenic effects (38), although this is thought to be dose and context dependent, in keeping with the pleiotropism that can characterize cytokines. In our studies and previous reports (12, 39), DR-18 has not displayed tumor-promoting activity. Expression of decoy-resistant IL-18 variants is also being utilized in adoptive cell therapies, including chimeric antigen receptor T cells, to potentiate antitumor effects, and it has shown promising preclinical activity (40).

This study has several limitations. The predictive biomarker studies on IL-18BP relied on small, single-institution RCC cohorts. Further work in larger, multiinstitution cohorts is needed to verify these findings. Additionally, although Renca and RAG are well-established murine RCC models, neither cell line mimics human RCC genetics. As a result, their clinical predictive value may be more limited. While the field was previously constrained by the lack of other syngeneic, immunocompetent murine models, a potentially novel syngeneic murine RCC cell line, LVRCC67, was developed recently by engineering the loss of *Vhl*, *p53*, and *Rb1* and the overexpression of *c-myc* (41). Future studies should incorporate these and other models into preclinical testing.

Despite these caveats, the results of this study still strongly suggest that a combination of the human version of DR-18 with an anti–CTLA-4 agent may be an effective treatment option in RCC. Currently, the best treatment strategy at the time of progression with ICI-resistant RCC is unclear. Various VEGF pathway–targeting drugs are commonly used, with response rates in the approximately 20%–45% range, although with limited duration of responses and very few if any long-term responses (PFS of 6–12 months) (42–47). The combination of atezolizumab, an anti–PD-L1 agent, with cabozantinib offered no additional benefit over cabozantinib alone in the ICI-resistant/refractory setting (48). Additionally, patients with RCC treated with an anti–CTLA-4–containing regimen after nonresponse to an anti–PD-1–containing regimen have overall response rates in the 4%–15% range across historical studies (49–52). Our human transcriptomic, qIF, and ELISA findings in RCC suggest that the IL-18 pathway may be poised for reactivation in RCC with an agent like DR-18 that can bypass the inhibitory protein IL-18BP, particularly in ICI-nonresponding patients. Given these data, the efficacy of DR-18 and anti–CTLA-4 combined therapy in the models tested, including Renca, a relatively ICI-resistant model, and prior findings on DR-18 efficacy in the MHC-I–deficient setting (12), a clinical trial exploring the safety and efficacy of DR-18 + anti–CTLA-4 in ICI-resistant/refractory RCC should be considered, potentially exploring changes in IL-18BP levels in tumor and/or plasma to select patients.

## Methods

### Sex as a biological variable.

For studies involving patient specimens, specimens from both male and female patients were included, reflecting the underlying sex ratio of RCC (roughly 2:1 male/female ratio). For the mouse studies, only male mice were used, as the mouse cancer cell lines used in this study derived from male mice only.

### Patient specimens.

Human plasma samples were collected at Yale University (New Haven, Connecticut, USA) from patients with RCC treated with ICI-containing regimens. Samples used for analysis were collected at baseline (pretreatment) and at the beginning of the third cycle of treatment for nearly all patients (approximately 6 weeks later). Patient and tumor characteristics, treatment responses, and time points of sample collection are noted in Supplemental Table 4.

### Mice.

BALB/cJ-000651 and C57BL/6J-000664 mice were ordered from The Jackson Laboratory and used in the indicated experiments. Age- and sex-matched mice were used.

### Cell lines.

The following cell lines were used: Renca (ATCC, CRL-2947), RAG (ATCC, CCL-142), and YUMMER1.7 (Yale University, M. Bosenberg) (53). Renca cells were cultured in RPMI-1640 (Corning, 10-040-CV) plus 10% FBS (Thermo Fisher Scientific, 16140-071), 1× MEM nonessential amino acids (Thermo Fisher Scientific, 11140-050), sodium pyruvate (1 mM) (Thermo Fisher Scientific, 11360-070), L-glutamine (2 mM) (Thermo Fisher Scientific, 25030-081), and 1× antibiotic-antimycotic (Thermo Fisher Scientific, 15240-062). RAG cells were grown in EMEM with 1.5 g/L sodium bicarbonate, nonessential amino acids, L-glutamine, and sodium pyruvate (Corning, 10-009-CV) plus 10% FBS and 1× antibiotic-antimycotic. YUMMER1.7 cells were cultured in DMEM/F12 with L-glutamine and 15 mM Hepes (Thermo Fisher Scientific, 11330-032) plus 10% FBS, 1× MEM nonessential amino acids, and 1× antibiotic-antimycotic. All cells were cultured at 37°C, 5% CO_2_, and kept at low passage prior to mouse engraftment (less than passage 10–12).

Mycoplasma testing was performed using the MycoAlert Mycoplasma Detection Kit (Lonza, LT07-318); all cell lines tested negative.

### IF staining.

Two previously reported RCC TMAs were used for IL-18BP qIF analysis: YTMA166, containing paired primary tumors and metastases, and YTMA-528, containing primary tumors and metastases, including brain metastases, from brain-metastases susceptible patients (13, 14, 54–56). The TMAs consisted of 0.6 mm cores spaced 0.8 mm apart. Two independent pathologists had reviewed and selected areas of tumor. Collection of patient specimens and clinical data was approved by the Yale University IRB. Characteristics of the tumor specimens included for analysis are shown in Supplemental Table 3.

IF staining of the 2 TMAs was performed as previously described (57, 58). Briefly, 5 μm TMA sections mounted on glass slides were deparaffinized in xylene, rinsed in ethanol, and then boiled for 15 minutes in 6.5 mM citrate buffer (pH 6.0) for antigen retrieval. Slides were then incubated with methanol and 0.75% hydrogen peroxide, blocked with 0.3% bovine serum albumin (BSA) in TBS, and incubated overnight at 4°C with anti–IL-18BP antibody (Invitrogen, PA5-116465) diluted 1:800 in 0.3% BSA/TBS. A signal amplification step was added using the secondary anti-rabbit EnVision antibody + HRP (Dako, K4003) and HRP-activated Cy5-tyramide (1:50; Akoya Biosciences, SAT705A001EA) following the manufacturer’s protocol. HRP quenching was performed with 100 mM benzoic hydrazide + 50 mM hydrogen peroxide in PBS. Following washings, to create a tumor mask, slides were incubated overnight at 4°C with anti-CA9 (1:1,000; gift of Jan Zavada) and anti-cytokeratin (1:200; Dako, M3515) antibodies and streptavidin HRP (1:200; MilliporeSigma, S2438) in 0.3% BSA/TBS. Slides were washed and the signal was amplified using anti–mouse EnVision system + HRP (Dako K4001) and Cy3-Tyramide (1:50; Akoya Biosciences, SAT704A001EA). Slides were incubated for 20 minutes with DAPI diluted at 1:300 and mounted with ProLong Gold Antifade Mountant (Invitrogen, P36931).

### Multispectral image acquisition and quantitative determination of target expression.

Image acquisition and quantitative measurements were performed as previously described (57). The tumor mask was created from the CA9/cytokeratin signal through automated processing and thresholding and was used to distinguish tumor from stromal elements. A total tissue mask (tumor plus stroma) was created from the DAPI signal, which defined the nuclear compartment. A stromal compartment was created by subtracting the tumor mask from the total tissue mask. IL-18BP signal (total normalized signal intensity/area of the compartment) was quantified for the tumor and stromal compartments and was then summed for the total IL-18BP signal in the TME (tumor + stroma). Tumor spots were excluded if they contained insufficient tissue or abundant necrotic tissue or if they contained significant artifacts.

### ELISA.

IL-18 and IL-18BP ELISAs were performed using the Human Total IL-18 DuoSet ELISA (R&D Systems, DY318-05), DuoSet ELISA Ancillary Reagent Kit 2 (R&D Systems, DY008B), and human IL-18BP ELISA Kit (Abcam, ab100559) according to the manufacturers’ instructions.

### Tumor treatment studies.

Tumor cells were engrafted s.c. onto the flanks of 7- to 9-week-old age-matched male mice. The following number of tumor cells were engrafted per mouse: 0.5 × 10^6^ Renca cells, 1.0 × 10^6^ RAG cells, and 0.5 × 10^6^ YUMMER1.7 cells. Drug treatment was started when the mean tumor size was between 50 and 100 mm^3^ (usually at day 7 after engraftment for Renca and YUMMER1.7 tumors, and day 10 for RAG tumors); mice with tumors less than 30 mm^3^ or greater than 150 mm^3^ at this time were excluded from treatment. The remaining mice were randomized into treatment groups and treated twice weekly for 5 doses for the efficacy studies and for 3 doses for the cytokine/chemokine and single-cell transcriptomic profiling studies. Antibody treatments were delivered i.p., and DR-18 was delivered s.c. Drug treatments were diluted in sterile PBS and dosed as follows: anti–PD-1 (clone RMP1-14, BioXCell, BE0146) 200 μg; anti–CTLA-4 (clone 9H10, BioXCell, BE0131) 200 μg for Renca, RAG tumors, and 50 μg for YUMMER1.7 tumors; and DR-18 0.32 mg/kg. Control groups were treated with sterile PBS. Tumor growth was monitored at least twice weekly by caliper measurement. Tumor volumes were calculated as follows: volume = 0.5233 × length × width × height. Mice were euthanized when tumors reached IACUC-approved endpoints (volume greater than or equal to 1,000 mm^3^ or ulceration). Survival analyses reflect these endpoints. The investigators were not blinded to the treatment allocation during experiments and outcome assessment.

For the immune cell depletion/effector molecule neutralization studies, depleting/neutralizing antibodies were injected 24 hours prior to each drug treatment (including the first drug treatment) and then twice weekly for the duration of the experiment. The following depleting/neutralizing antibodies were used: anti-CD8a (clone 2.43, BioXCell, BE0061); anti-CD4 (clone GK1.5, BioXCell, BE003-1); and anti–IFN-γ (clone XMG1.2, BioXCell, BE0055). For NK cell depletion, anti–Asialo GM1 (clone Poly21460, BioLegend, 146002) was used. Anti-CD8a, anti-CD4, and anti–IFN-γ were given i.p. at 200 μg/mouse. Anti–Asialo GM1 was reconstituted in 1 mL PBS, and 50 μL of a 1:2.5 dilution in PBS was given i.p.

For tumor rechallenge studies, mice with complete RAG or YUMMER1.7 tumor regression were reinoculated s.c. with twice the initial dose of tumor cells (2.0 × 10^6^ RAG cells; 1.0 × 10^6^ YUMMER1.7 cells) at day 100 after initial tumor cell engraftment. Tumor growth and survival were monitored twice weekly as above for 60 days, although no tumors grew out on rechallenge.

### Mouse cytokine/chemokine profiling.

Whole blood was collected retro-orbitally from mice 24 hours after the first treatment and upon euthanasia 24 hours after the third treatment. Plasma was isolated and cytokine/chemokine profiling was performed using the 31-plex Mouse Cytokine/Chemokine Array from Eve Technologies (MD31).

### scRNA-Seq sample preparation.

Using the same mice as above for cytokine/chemokine profiling, with 3 mice per treatment group, mice were euthanized and tumors were harvested for analysis 24 hours after the third treatment. Tumors were dissociated by mincing in RPMI + 2% FBS, incubating with 0.1 mg/mL collagenase and DNase I for 30 minutes at 37°C, and filtering through a 70 μM filter to obtain a single cell suspension. They were then washed with RPMI + 10% FBS and resuspended in RPMI + 20% FBS. For sorting, cells were incubated for 30 minutes at 4°C with fluorophore-conjugated antibodies using the following antibodies: anti-CD45 (clone 30-F11, BD Biosciences) and anti-CD3 (clone 17A2, BD Bioscience). Samples were sorted using a BD FACSAriaII into 3 populations: T cells (CD45^+^CD3^+^); non–T immune cells (CD45^+^CD3^–^); and tumor and stromal cells (CD45^–^CD3^–^). For live/dead staining, AmCyan Kit (Thermo Fisher Scientific) was used. Sorted cells for each subset were counted manually and then combined in a 2:1:1 ratio of T cells/non-T immune cells/tumor and stromal cells, with an equal contribution from each biologic replicate from an experimental condition. Ten thousand cells from each of the mixed sorted samples for each condition were loaded onto the 10X Genomics Chromium System. Library preparation for scRNA-Seq and scTCR-Seq was performed using the 5′ Reagent Kit from 10X Genomics according to the manufacturer’s instructions by the Yale Center for Genome Analysis (YCGA) and passed quality control. Libraries were sequenced using an Illumina NovaSeq (1 library per lane) at the YCGA.

### scRNA-Seq analysis.

CellRanger was used to align reads to the mouse reference transcriptome (mm10) and to generate cell-by-gene matrices for each sample library. The Seurat package for R v4.3.0 was used to process the matrices and perform downstream analysis. Low quality cells were filtered out that did not meet the following thresholds: ≥ 500 nUMI; ≥ 250 genes; > 0.785 log_10_GeneperUMI; and < 0.3 mitochondrial gene ratio. Genes expressed in fewer than 10 cells were also filtered out. Cell cycle scoring was performed using the CellCycleScoring command using mouse gene sets orthologous to previously described human gene sets. Cell cycle factors were regressed out using the SCTransform function, and the data were normalized and integrated on the 3,000 most variable features. Principal component (PC) scores from the first 40 PCs were used for clustering with the FindClusters command, and a resolution of 0.8. Uniform Manifold Approximation and Projection (UMAP) was used for dimensionality reduction. Cell type assignments for each cluster were performed using SingleR (59) and mouse cell reference datasets (and the ZilionisLungData for mouse for the neutrophil subtype analysis; ref. 25) and verified with expression patterns of cell-type defining markers (Supplemental Figure 5C) and examination of the top 10 conserved markers per cluster (from the FindConservedMarkers function). Clusters identified as stressed or dying cells or with clear mixed immune cell populations, which only comprised clusters with a small total number of cells, were removed from further analysis with the Subset command. Gene expression UMAP plots were generated using the FeaturePlot command. Cluster frequencies by experimental condition were normalized to the total number of cells per condition. The top differentially expressed genes comparing a single cluster to all other clusters were computed using the FindAllMarkers function, the data were scaled, and heatmaps of the top differentially expressed genes by adjusted *P* value were created from the Pheatmap package and the DoHeatMap function. Dot plots were generated from DotPlot command. Enhanced Volcano plots were generated from the EnhancedVolcano package. Gene set enrichment analysis was performed using EnrichR. T cells, macrophages/monocytes, and neutrophils were further subsetted and analyzed separately as described above, with additional analysis as below.

T cell subsets were clustered as above but were additionally annotated using ProjecTILS and a reference mouse tumor-infiltrating T cell dataset (60). Milo was used for differential abundance analysis using K nearest neighbor analysis (17), with k = 30, d = 30, and prop = 0.1 for the buildGraph and makeNhoods functions, d = 30 for the calcNhoodDistance function, and anti–CTLA-4 treatment status set as a covariate. Data were log normalized and aggregated by experimental condition, and automatic grouping of neighborhoods was performed. For T cell subset analysis, max.lfc.delta = 1.75 and overlap = 5 using the groupNhoods function; for neutrophil subset analysis, max.lfc.delta = 1.5 and overlap = 0. To compare neighborhood groups, the findNhodGroupMarkers function was used.

TCR analysis was performed using scRepertoire package (61) and the filtered contig annotations. NicheNet (26) analysis was performed using the mouse ligand-receptor network and ligand-target matrix, the granulocyte clusters were set as the “receiver,” the macrophage, monocyte, CD8^+^, CD4^+^, Tregs, and NK cell clusters were set as the “sender”, and there was a lfc_cutoff=0.15. Pseudotime analysis was performed using the Slingshot package, “UMAP” was set for dimensionality reduction, and the control condition (“PBS”) was specified as the starting point (62).

### Analysis of TCGA data.

TCGA PanCancer Atlas data were accessed from the cBioPortal (63, 64) and analyzed using the web browser and in R. For PanCancer analysis, RNASeqV2 RSEM processed and normalized data were used (which corresponds to the rsem.genes.normalized_results file from TCGA). For RCC-specific analysis, mRNA expression *Z* scores were used, with the reference population set to normal samples. See the cBioPortal User Guide for more information on the RNA data available. For *IL18BP* analysis with ccRCC, patient samples were dichotomized based on the median mRNA *Z* score.

### Statistics.

Statistical analyses were conducted using R v4.2.2 and Prism 9 (GraphPad Software), and the statistical tests are specified in the text and figure legends. Briefly, the Kruskal-Wallis test with Dunn’s correction for multiple comparisons was used for testing between more than 2 groups; the Mann-Whitney *U* test for comparisons between 2 groups; Wilcoxon matched-pairs signed rank test for matched-pair comparisons; the Fisher’s exact test for comparisons of the proportions of 2-group variables; the χ^2^ test for comparison of the distribution of multiple categories; and the log-rank test for comparison of Kaplan-Meier survival curves. Generally, corrected *P* < 0.05 were considered significant.

### Study approval.

The patient specimen study protocols were approved by the IRB of Yale University, and all patients provided written informed consent. Mice were maintained in accordance with the guidelines from the Yale University IACUC. Mouse experiments were performed in accordance with IACUC-approved protocols using and age- and sex-matched mice.

### Data availability.

scRNA-Seq and TCR-Seq data have been deposited on the public database GEO (accession no. GSE279662). Other data are available in the Supportive Data Values file or from the corresponding authors upon request.

## Author contributions

DAS, HMK, and AMR participated in conceptualization. DAS, HMK, AMR, BYL, DAB, MH, and LJ assisted in methodology. DAS, DD, DGS, LZ, LK, JEM, and JDH performed investigation. DAS and HMK participated in visualization, funding acquisition, project administration, and writing (original draft and review/editing/revisions). HMK and AMR performed supervision.

## Supplementary Material

Supplemental data

Supplemental tables 1-4

Supporting data values

## Version 1 11/19/2024

In-Press Preview

## Version 2 01/09/2025

Electronic publication
