# Supplementary material for: Decoy-resistant IL-18 reshapes the tumor microenvironment and enhances rejection by anti–CTLA-4 in renal cell carcinoma
Source: JCI Insight. 2024 Nov 19;10(1):e184545. doi: 10.1172/jci.insight.184545 (PMC11721305; doi:10.1172/jci.insight.184545)
Supplement: Supplemental data [file jciinsight-10-184545-s163.pdf]

Supplementary Information:

Supplementary Figure Legends:

Supp. Fig. S1: ccRCC has high expression levels of *IL18BP, IL18R1, and IL18RAP*. A. Expression of the indicated transcript across cancer types from TCGA PanCancer Atlas, ordered by increasing median value. ccRCC (*) and non-ccRCC (*) are indicated with red and blue stars, respectively. *IL18RAP* expression from TCGA PanCancer Atlas for **(B)** RCC histologic subtypes, and **(C)** for ccRCC, by stage. **D.** Tumor grade for ccRCC tumors from TCGA PanCancer Atlas, by *IL18BP* expression level. **E.** Indicated Hypoxia Score for ccRCC tumors from TCGA PanCancer Atlas, by *IL18BP* expression level. For (B-C), statistical testing was performed using Kruskal-Wallis test with Dunn's correction for multiple comparisons; for (D), a Chi-Squared test was performed; and for (E), a Wilcoxon test was used. * $P < 0.05$; ** $P < 0.01$; *** $P < 0.001$; **** $P < 0.0001$.

Supp. Fig. S2: In ccRCC, *IL18BP* expression is highly correlated with immune checkpoint expression. A. The top gene sets from enrichment analysis of transcripts enriched with low *IL18BP* expression, from ccRCC TCGA PanCancer database. **B.** Correlation of *IL18BP* expression with the indicated transcripts for immune checkpoints (*LAG3, TIGIT, PDCD1, CTLA4*), the Treg marker *FOXP3*, and *CD4*, which were all among the top increased transcripts with high *IL18BP* expression, and **(C)** correlation with *IL18*, from ccRCC TCGA PanCancer database. The Spearman's correlation r is shown.

Supp. Fig. S3: In RCC, IL-18BP protein levels increase post-ICI treatment in non-responding patients. A. Representative IL-18BP IF staining on RCC histospots, with exemplary patterns of Low, Medium, and High expression. **B.** IL-18BP protein levels in the RCC TME (tumor plus stroma) assessed by qIF, by anatomic location (EC met = extra-cranial metastases; Brain met = brain metastases). **C.** Circulating plasma levels of IL-18BP by treatment response, separated by time of treatment (before- and on- ipi + nivo). **D.** Circulating plasma levels of IL-18 from patient-matched samples pre- and post- ipi + nivo treatment. **E.** Circulating plasma levels of IL-18 from patient-matched samples before- and on- ipi + nivo treatment, separated by treatment response. Circulating plasma levels of IL-18 by treatment response, separated by time of treatment (before- and on- ipi + nivo) **(F)**, the ratio of post/pre-treatment levels by treatment response **(G)**, and directional change of levels post-treatment by response **(H)**. **I.** Correlation between circulating plasma levels of IL-18 and IL-18BP, by treatment response, separated by time of treatment (before- and on- ipi + nivo). Statistical testing was performed using Kruskal-Wallis test with Dunn's correction for multiple comparisons (B), Mann-Whitney test (C, F, G), Wilcoxon matched-pairs signed rank test (D-E), and Fisher's exact test (H). ns = non-significant; * $P < 0.05$; ** $P < 0.01$; *** $P < 0.001$; **** $P < 0.0001$.

Supp. Fig. S4: In RCC murine models, DR-18 plus anti-CTLA-4 has high anti-tumor activity. Spider plots of tumor growth with the indicated treatments in the Renca RCC model **(A)**, the RAG RCC tumor model **(B)**, and the YUMMER1.7 melanoma model **(F)**. The red line illustrates the tumor size endpoint, and the numbers on the graphs show the number of mice reaching endpoint over the total number of mice tested.

C-D. Kaplan-Meier survival curves and mean tumor growth curves (mean +/- standard error of the mean) of mice engrafted with RAG (select treatment groups shown for ease of viewing – from same experiments as in Fig. 3D-E). **E.** Kaplan-Meier survival curves of mice engrafted with YUMMER1.7. For Kaplan-Meier curves, statistical testing was performed using the log-rank test with Bonferroni correction in comparison to control-treated mice (C) or the corresponding single-agent ICI for the combination regimens (E). * $P < 0.05$; ** $P < 0.01$; *** $P < 0.001$; **** $P < 0.0001$.

Supp. Fig. S5: Single-cell transcriptomic landscape of Renca tumors treated with DR-18, anti-CLTA-4, or the combination. A. UMAP plot of clustering and annotation (labeled) of all cell populations isolated from Renca tumors treated for three cycles with PBS, DR-18, anti-CTLA-4, or DR-18 + anti-CTLA-4 ("Combo") (n=3 mice/group, pooled) based on scRNA-seq analysis. Annotations were performed using SingleR. **B.** Heatmap of the top differentially expressed genes between the cell populations identified in **(A)**. **C.** UMAP plots of all cells analyzed showing expression of genes supporting cell type assignments. **D.** Percentage of cells from each treatment group by cell type.

Supp. Fig. S6: Unsupervised quantitative analysis of T cell subsets using Milo. A. Neighborhood plot from Milo analysis of T cell subsets from scRNA-seq data, showing neighborhood fold-change (FC) values with DR-18 plus anti-CTLA-4 treatment. Volcano plots comparing gene expression in neighborhood group 7 to **(B)** neighborhood group 4 and **(C)** neighborhood group 1, from Milo analysis of T cell subsets. Heatmaps of the top differentially expressed genes between neighborhood group #7, enriched with DR-18 plus anti-CTLA-4 treatment, versus **(D)** neighborhood group #1, and **(E)** neighborhood group #5.

Supp. Fig. S7: T cell landscape in the TME of Renca tumors treated with DR-18, anti-CTLA-4, or the combination. UMAP plots of T cell subsets isolated from Renca tumors treated for three cycles with PBS, DR-18, anti-CTLA-4, or DR-18 plus anti-CTLA-4 ("Combo") (n=3 mice/group, pooled) based on scRNA-seq analysis, showing **(A)** unsupervised hierarchal clustering and **(B)** overlaid treatment groups. **C.** Heatmap of the top differentially expressed genes between the T cell clusters identified in **(A)**. **D.** UMAP plot of all T cells as in **(A-B)**, with overlaid phenotypic annotations of T cell subsets using ProjectTILS (CD8_Tpex = $CD8^+$ precursor exhausted; CD8_Tex = $CD8^+$ terminally exhausted). **E.** Radar plots comparing reference T cell phenotypic groups from ProjectTILs ("Reference") to the T cell subsets identified in **(D)** ("Query"). **F.** Percentage of T cell subsets identified in **(D)** by treatment group. For select cell populations (boxed), the percentages within each treatment group are shown. **G.** Percentage of cells from each treatment group by T cell subsets identified in **(D)**.

Supp. Fig. S8: scTCR-seq analysis reveals expansion of dominant $CD8^+$ effector clonotype with DR-18 plus anti-CTLA-4. A. Dotplot of immune checkpoint expression on T cells by treatment group, from scRNA-seq analysis. **B.** Relative proportion of each clonotype for the top 10 clonotypes by treatment group, from scTCR-seq analysis. **C.** Diversity analysis indices for each treatment group based on scTCR-seq analysis, showing lower clonal diversity (Shannon and Inv. Simpson indices) with DR-18 plus anti-CTLA-4 ("Combo"), higher richness (Chai index), and lower evenness (Inv. Pielou index). **D.** Alluvial plot showing expansion of

a dominant clonotype from DR-18 treatment to DR-18 plus anti-CTLA-4 treatment. **E.** Dotplot of the top differentially expressed genes between the dominant clonotype from **(D)** and the non-dominant clonotypes, showing a $CD8^+$ effector phenotype in the dominant clonotype.

Supp. Fig. S9: Myeloid cell landscape in the TME of Renca tumors treated with DR-18, anti-CTLA-4, or the combination. A. UMAP plot of macrophage/monocyte subsets isolated from Renca tumors treated for three cycles with PBS, DR-18, anti-CTLA-4, or DR-18 plus anti-CTLA-4 ("Combo") (n=3 mice/group, pooled) based on scRNA-seq analysis, showing unsupervised hierarchal clustering. **B.** Heatmap of the top differentially expressed genes between the macrophage/monocyte annotated clusters identified in Fig. 6B. **C.** Percentage of cells from each treatment group by macrophage/monocyte subsets shown in **(B)**. **D.** UMAP plot of granulocyte subsets isolated from Renca tumors treated for three cycles with PBS, DR-18, anti-CTLA-4, or DR-18 plus anti-CTLA-4 ("Combo") (n=3 mice/group, pooled) based on scRNA-seq analysis, showing unsupervised hierarchal clustering. **E.** Heatmap of the top differentially expressed genes between the neutrophil phenotypic subtypes shown in Fig. 6G. **F.** Percentage of cells from each treatment group by neutrophil subtypes shown in **(E)**.

Supp. Fig. S10: Myeloid cell landscape in the TME of Renca tumors treated with DR-18, anti-CTLA-4, or the combination. A. Granulocyte ligand-receptor network analysis, based on NicheNet analysis of scRNA-seq dataset. Neighborhood plot showing neighborhood fold-change (FC) values with DR-18 plus anti-CTLA-4 treatment ("Combo") **(B)**, neighborhood group plot **(C)**, and UMAP plot with annotated neutrophil phenotypic subtypes with overlaid treatment groups **(D)** from Milo analysis of granulocyte subsets from scRNA-seq data. **E.** Differential abundance fold changes of the neighborhood groups in **(C)**, comparing the Combo treatment to control, showing enrichment and de-enrichment of certain groups. **F.** Heatmap of the top differentially expressed genes between neighborhood group #4, enriched with DR-18 + anti-CTLA-4 treatment, versus neighborhood group #2, de-enriched with combination treatment.

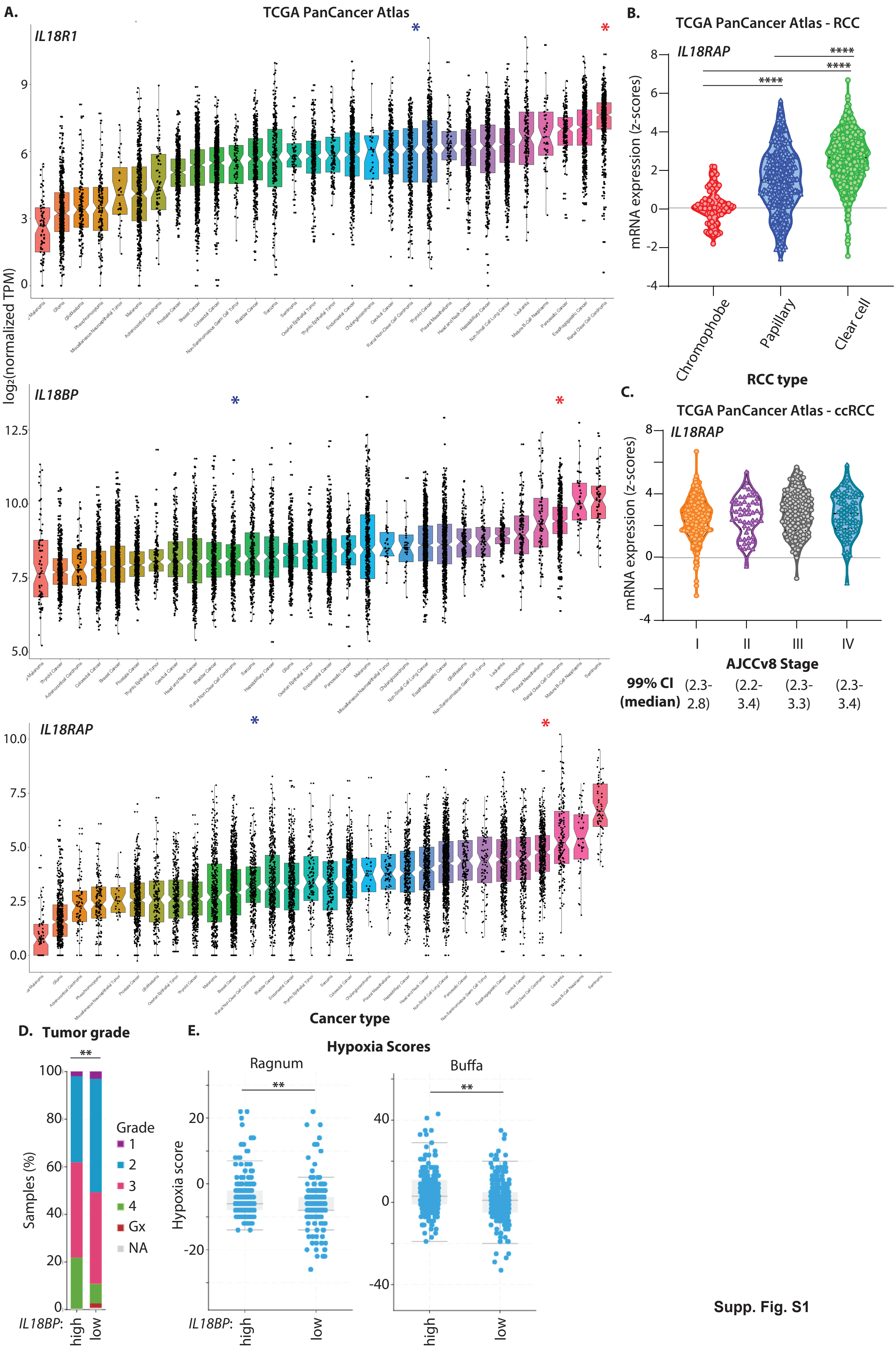
A.
TCGA PanCancer Atlas
IL18R1
IL18BP
IL18RAP
log2(normalized TPM)
Cancer type
B.
TCGA PanCancer Atlas - RCC
IL18RAP
****
****
****
mRNA expression (z-scores)
Chromophobe
Papillary
Clear cell
RCC type
C.
TCGA PanCancer Atlas - ccRCC
IL18RAP
mRNA expression (z-scores)
I
II
III
IV
AJCCv8 Stage
99% CI (median)
(2.3-2.8)
(2.2-3.4)
(2.3-3.3)
(2.3-3.4)
D. Tumor grade
**
Samples (%)
Grade
1
2
3
4
Gx
NA
IL18BP:
high
low
E.
Hypoxia Scores
Ragnum
Buffa
**
**
Hypoxia score
IL18BP:
high
low
high
low

Supp. Fig. S1

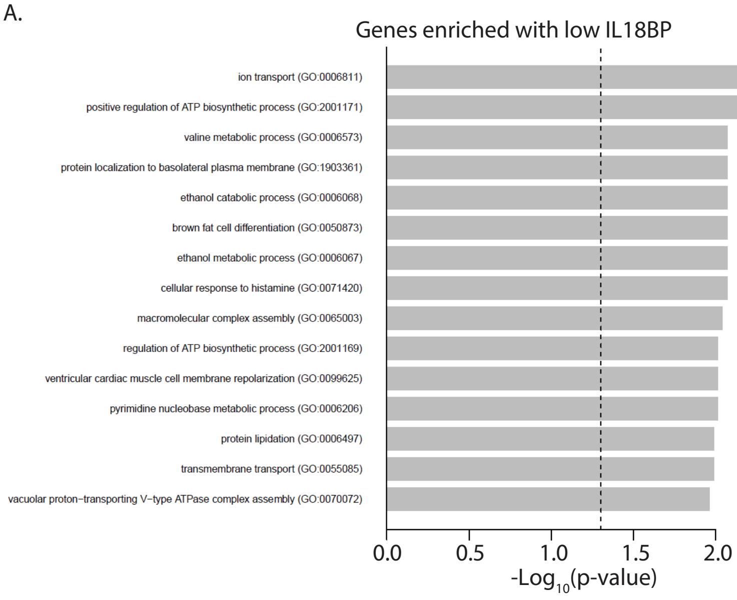
A.
Genes enriched with low IL18BP
ion transport (GO:0006811)
positive regulation of ATP biosynthetic process (GO:2001171)
valine metabolic process (GO:0006573)
protein localization to basolateral plasma membrane (GO:1903361)
ethanol catabolic process (GO:0006068)
brown fat cell differentiation (GO:0050873)
ethanol metabolic process (GO:0006067)
cellular response to histamine (GO:0071420)
macromolecular complex assembly (GO:0065003)
regulation of ATP biosynthetic process (GO:2001169)
ventricular cardiac muscle cell membrane repolarization (GO:0099625)
pyrimidine nucleobase metabolic process (GO:0006206)
protein lipidation (GO:0006497)
transmembrane transport (GO:0055085)
vacuolar proton−transporting V−type ATPase complex assembly (GO:0070072)
0.0
0.5
1.0
1.5
2.0
-Log10(p-value)

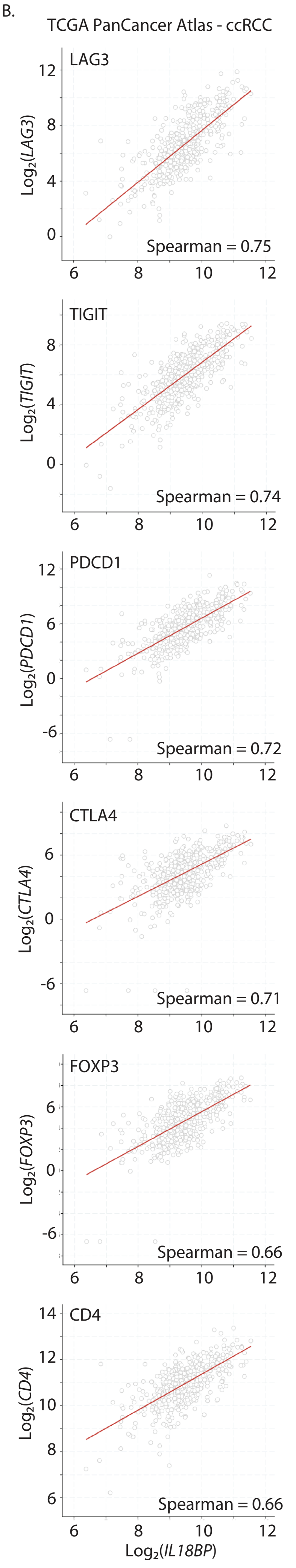
B.
TCGA PanCancer Atlas - ccRCC
LAG3
Log2(LAG3)
Spearman = 0.75
TIGIT
Log2(TIGIT)
Spearman = 0.74
PDCD1
Log2(PDCD1)
Spearman = 0.72
CTLA4
Log2(CTLA4)
Spearman = 0.71
FOXP3
Log2(FOXP3)
Spearman = 0.66
CD4
Log2(CD4)
Spearman = 0.66
Log2(IL18BP)

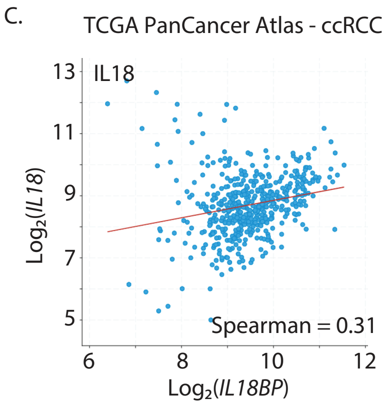
C.
TCGA PanCancer Atlas - ccRCC
IL18
Log2(IL18)
Spearman = 0.31
Log2(IL18BP)

Supp. Fig. S2

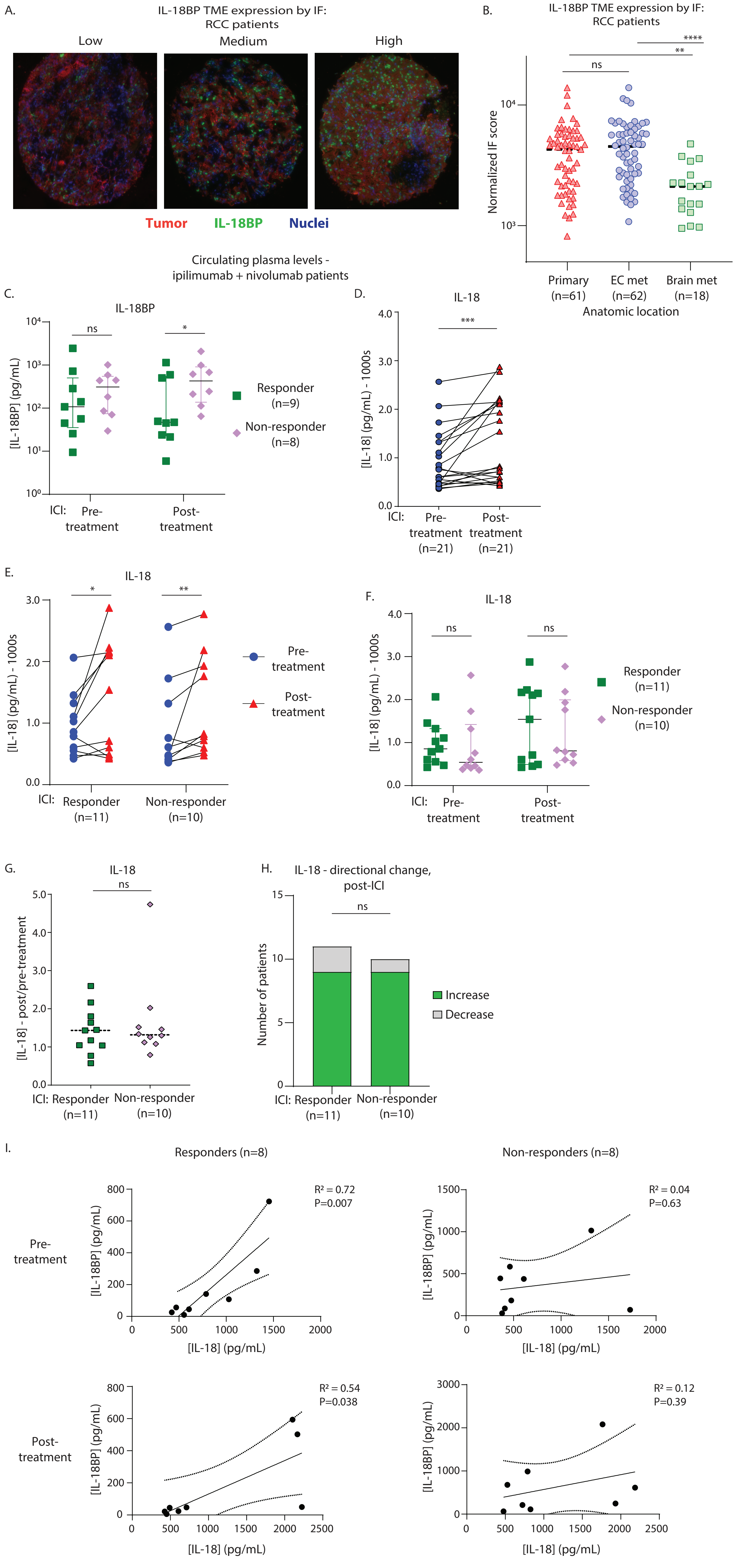
A.
IL-18BP TME expression by IF:
RCC patients
Low
Medium
High
Tumor
IL-18BP
Nuclei
B.
IL-18BP TME expression by IF:
RCC patients
****
**
ns
Normalized IF score
Primary
(n=61)
EC met
(n=62)
Brain met
(n=18)
Anatomic location
Circulating plasma levels -
ipilimumab + nivolumab patients
C.
IL-18BP
[IL-18BP] (pg/mL)
ns
*
Responder
(n=9)
Non-responder
(n=8)
ICI:
Pre-
treatment
Post-
treatment
D.
IL-18
***
[IL-18] (pg/mL) - 1000s
ICI:
Pre-
treatment
(n=21)
Post-
treatment
(n=21)
E.
IL-18
*
**
[IL-18] (pg/mL) - 1000s
Pre-
treatment
Post-
treatment
ICI:
Responder
(n=11)
Non-responder
(n=10)
F.
IL-18
ns
ns
[IL-18] (pg/mL) - 1000s
Responder
(n=11)
Non-responder
(n=10)
ICI:
Pre-
treatment
Post-
treatment
G.
IL-18
ns
[IL-18] - post/pre-treatment
ICI:
Responder
(n=11)
Non-responder
(n=10)
H.
IL-18 - directional change,
post-ICI
ns
Number of patients
Increase
Decrease
ICI:
Responder
(n=11)
Non-responder
(n=10)
I.
Responders (n=8)
Non-responders (n=8)
Pre-
treatment
Post-
treatment
[IL-18BP] (pg/mL)
[IL-18] (pg/mL)
R² = 0.72
P=0.007
R² = 0.04
P=0.63
R² = 0.54
P=0.038
R² = 0.12
P=0.39

Supp. Fig. S3

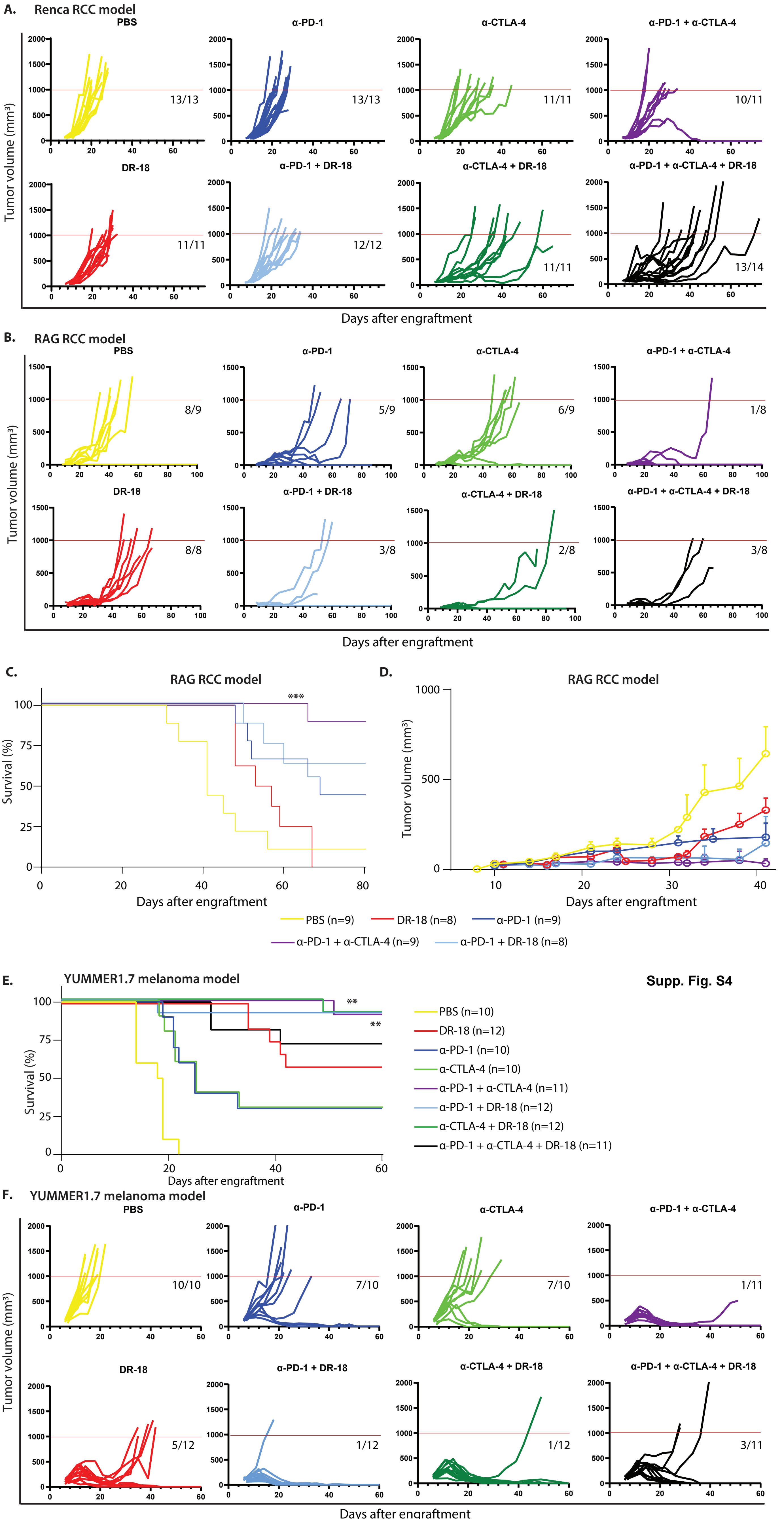
A. Renca RCC model
PBS
α-PD-1
α-CTLA-4
α-PD-1 + α-CTLA-4
DR-18
α-PD-1 + DR-18
α-CTLA-4 + DR-18
α-PD-1 + α-CTLA-4 + DR-18
13/13
13/13
11/11
10/11
11/11
12/12
11/11
13/14
Tumor volume (mm³)
Days after engraftment
B. RAG RCC model
PBS
α-PD-1
α-CTLA-4
α-PD-1 + α-CTLA-4
DR-18
α-PD-1 + DR-18
α-CTLA-4 + DR-18
α-PD-1 + α-CTLA-4 + DR-18
8/9
5/9
6/9
1/8
8/8
3/8
2/8
3/8
Tumor volume (mm³)
Days after engraftment
C. RAG RCC model
***
Survival (%)
Days after engraftment
D. RAG RCC model
Tumor volume (mm³)
Days after engraftment
PBS (n=9)
DR-18 (n=8)
α-PD-1 (n=9)
α-PD-1 + α-CTLA-4 (n=9)
α-PD-1 + DR-18 (n=8)
E. YUMMER1.7 melanoma model
**
**
Survival (%)
Days after engraftment
PBS (n=10)
DR-18 (n=12)
α-PD-1 (n=10)
α-CTLA-4 (n=10)
α-PD-1 + α-CTLA-4 (n=11)
α-PD-1 + DR-18 (n=12)
α-CTLA-4 + DR-18 (n=12)
α-PD-1 + α-CTLA-4 + DR-18 (n=11)
F. YUMMER1.7 melanoma model
PBS
α-PD-1
α-CTLA-4
α-PD-1 + α-CTLA-4
DR-18
α-PD-1 + DR-18
α-CTLA-4 + DR-18
α-PD-1 + α-CTLA-4 + DR-18
10/10
7/10
7/10
1/11
5/12
1/12
1/12
3/11
Tumor volume (mm³)
Days after engraftment

Supp. Fig. S4

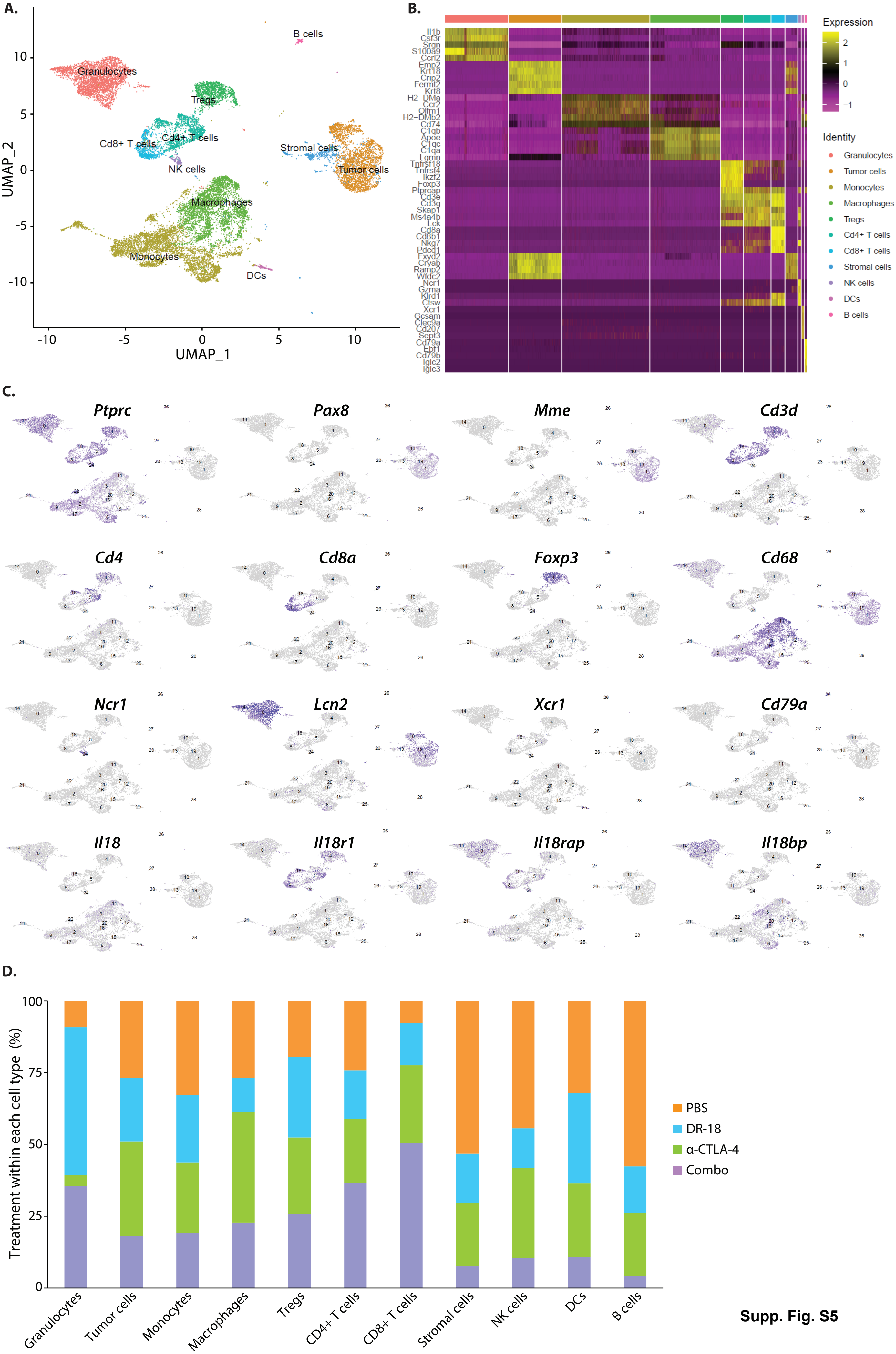
A.
B cells
Granulocytes
Tregs
Cd8+ T cells
Cd4+ T cells
NK cells
Stromal cells
Tumor cells
Macrophages
Monocytes
DCs
UMAP_1
UMAP_2
B.
Expression
Identity
Granulocytes
Tumor cells
Monocytes
Macrophages
Tregs
Cd4+ T cells
Cd8+ T cells
Stromal cells
NK cells
DCs
B cells
Il1b
Csf3r
Srgn
S100a9
Ccrl2
Emp2
Krt18
Crip2
Fermt2
Krt8
H2−DMa
Ccr2
Olfm1
H2−DMb2
Cd74
C1qb
Apoe
C1qc
C1qa
Lgmn
Tnfrsf18
Tnfrsf4
Ikzf2
Foxp3
Ptprcap
Cd3e
Cd3g
Skap1
Ms4a4b
Lck
Cd8a
Cd8b1
Nkg7
Pdcd1
Fxyd2
Cryab
Ramp2
Wfdc2
Ncr1
Gzma
Klrd1
Ctsw
Xcr1
Gcsam
Clec9a
Cd207
Sept3
Cd79a
Ebf1
Cd79b
Iglc2
Iglc3
C.
Ptprc
Pax8
Mme
Cd3d
Cd4
Cd8a
Foxp3
Cd68
Ncr1
Lcn2
Xcr1
Cd79a
Il18
Il18r1
Il18rap
Il18bp
D.
Treatment within each cell type (%)
PBS
DR-18
α-CTLA-4
Combo
Granulocytes
Tumor cells
Monocytes
Macrophages
Tregs
CD4+ T cells
CD8+ T cells
Stromal cells
NK cells
DCs
B cells

Supp. Fig. S5

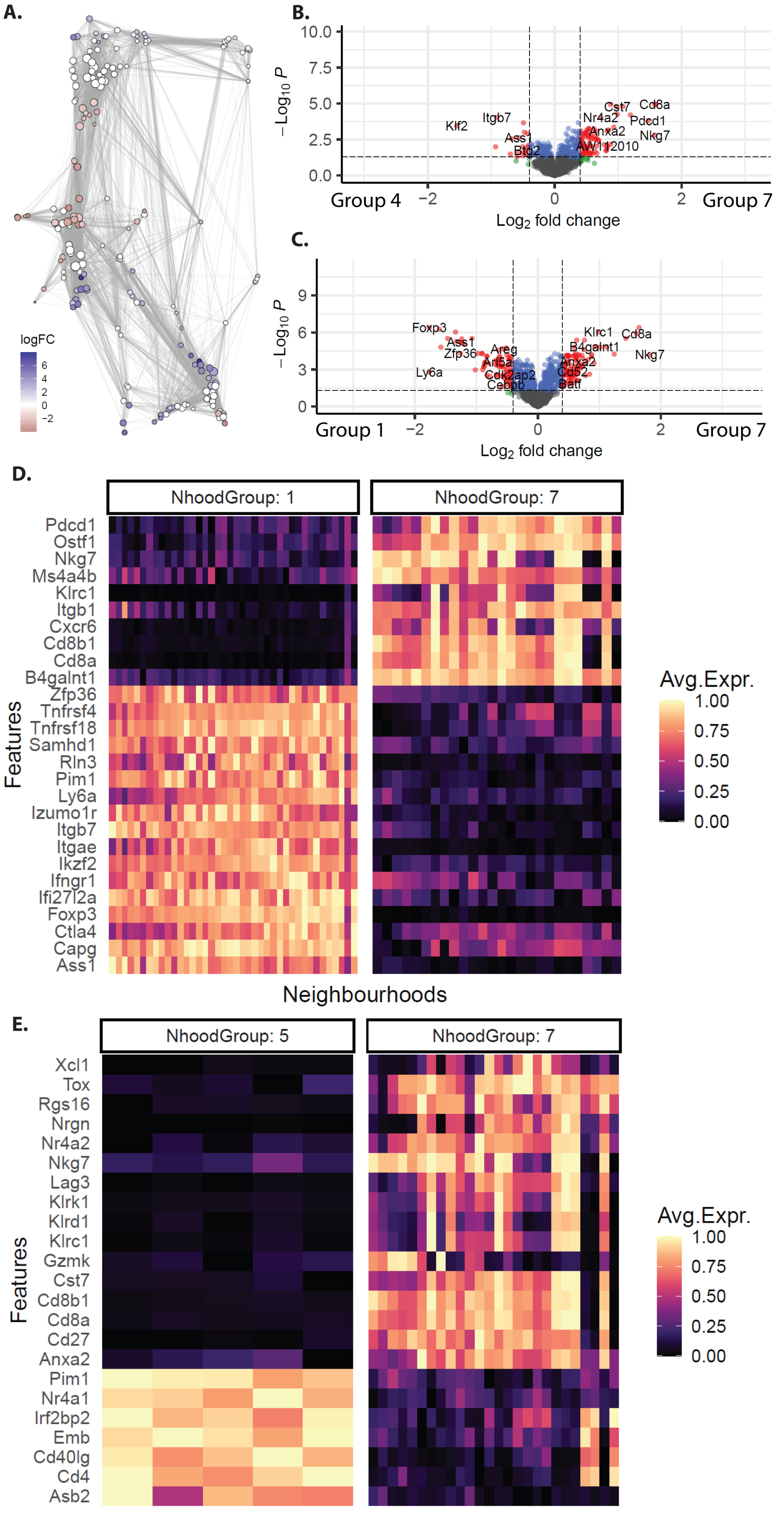

A.
logFC
6
4
2
0
−2
B.
10.0
7.5
5.0
2.5
0.0
−Log10 P
Cst7
Cd8a
Nr4a2
Pdcd1
Itgb7
Klf2
Anxa2
Ass1
Nkg7
Btg2
AW112010
Group 4
−2
0
2
Group 7
Log2 fold change
C.
9
6
3
0
−Log10 P
Foxp3
Klrc1
Cd8a
Ass1
B4galnt1
Zfp36
Areg
Nkg7
Arl5a
Anxa2
Ly6a
Cd52
Cdk2ap2
Cebpb
Batf
Group 1
−2
0
2
Group 7
Log2 fold change
D.
NhoodGroup: 1
NhoodGroup: 7
Pdcd1
Ostf1
Nkg7
Ms4a4b
Klrc1
Itgb1
Cxcr6
Cd8b1
Cd8a
B4galnt1
Zfp36
Tnfrsf4
Tnfrsf18
Samhd1
Rln3
Pim1
Ly6a
Izumo1r
Itgb7
Itgae
Ikzf2
Ifngr1
Ifi27l2a
Foxp3
Ctla4
Capg
Ass1
Features
Neighbourhoods
Avg.Expr.
1.00
0.75
0.50
0.25
0.00
E.
NhoodGroup: 5
NhoodGroup: 7
Xcl1
Tox
Rgs16
Nrgn
Nr4a2
Nkg7
Lag3
Klrk1
Klrd1
Klrc1
Gzmk
Cst7
Cd8b1
Cd8a
Cd27
Anxa2
Pim1
Nr4a1
Irf2bp2
Emb
Cd40lg
Cd4
Asb2
Features
Neighbourhoods
Avg.Expr.
1.00
0.75
0.50
0.25
0.00

Supp. Fig. S6

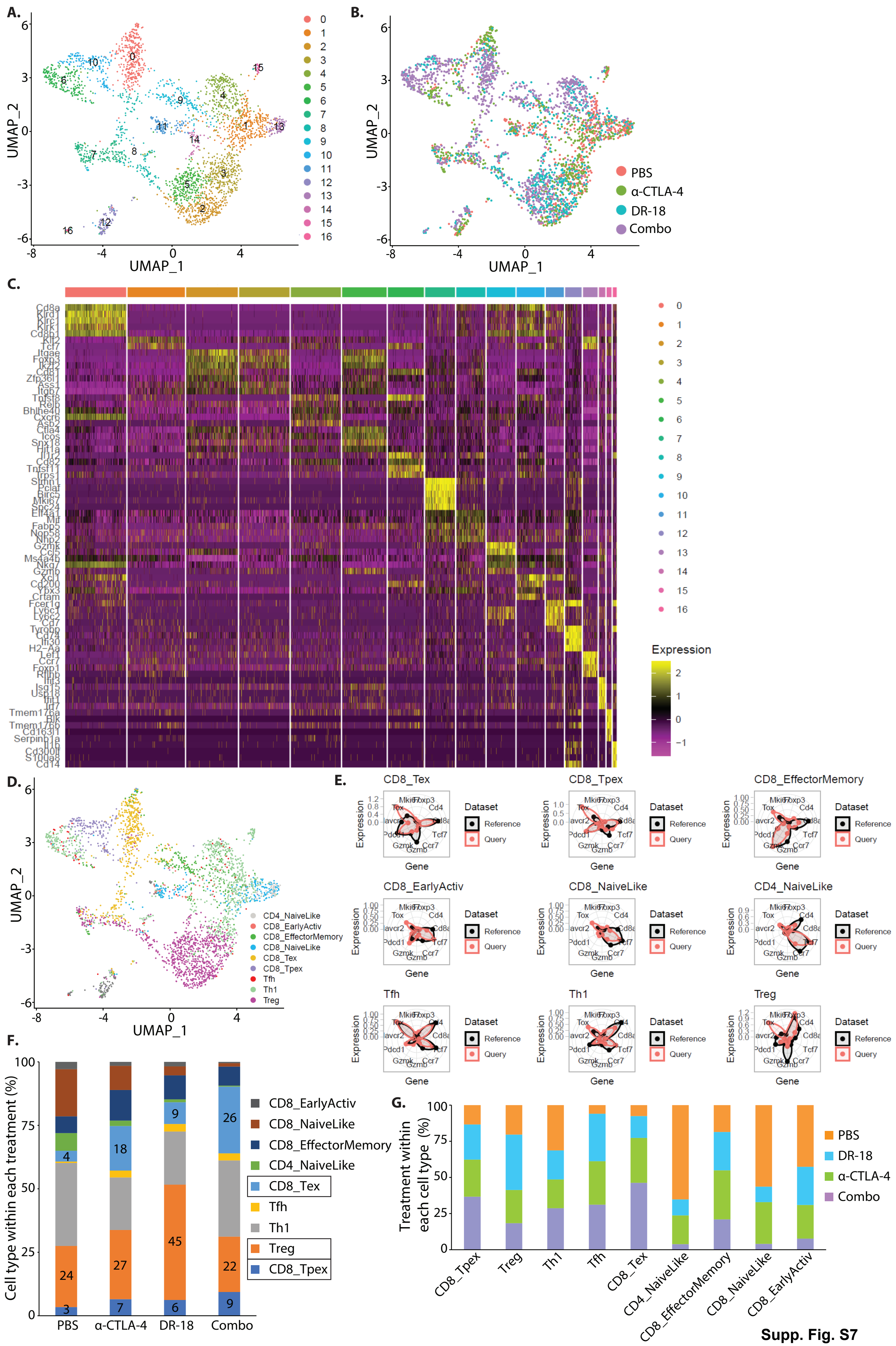
A.
B.
C.
D.
E.
F.
G.
UMAP_1
UMAP_2
PBS
α-CTLA-4
DR-18
Combo
Expression
CD4_NaiveLike
CD8_EarlyActiv
CD8_EffectorMemory
CD8_NaiveLike
CD8_Tex
CD8_Tpex
Tfh
Th1
Treg
Dataset
Reference
Query
Gene
Cell type within each treatment (%)
Treatment within each cell type (%)

Supp. Fig. S7

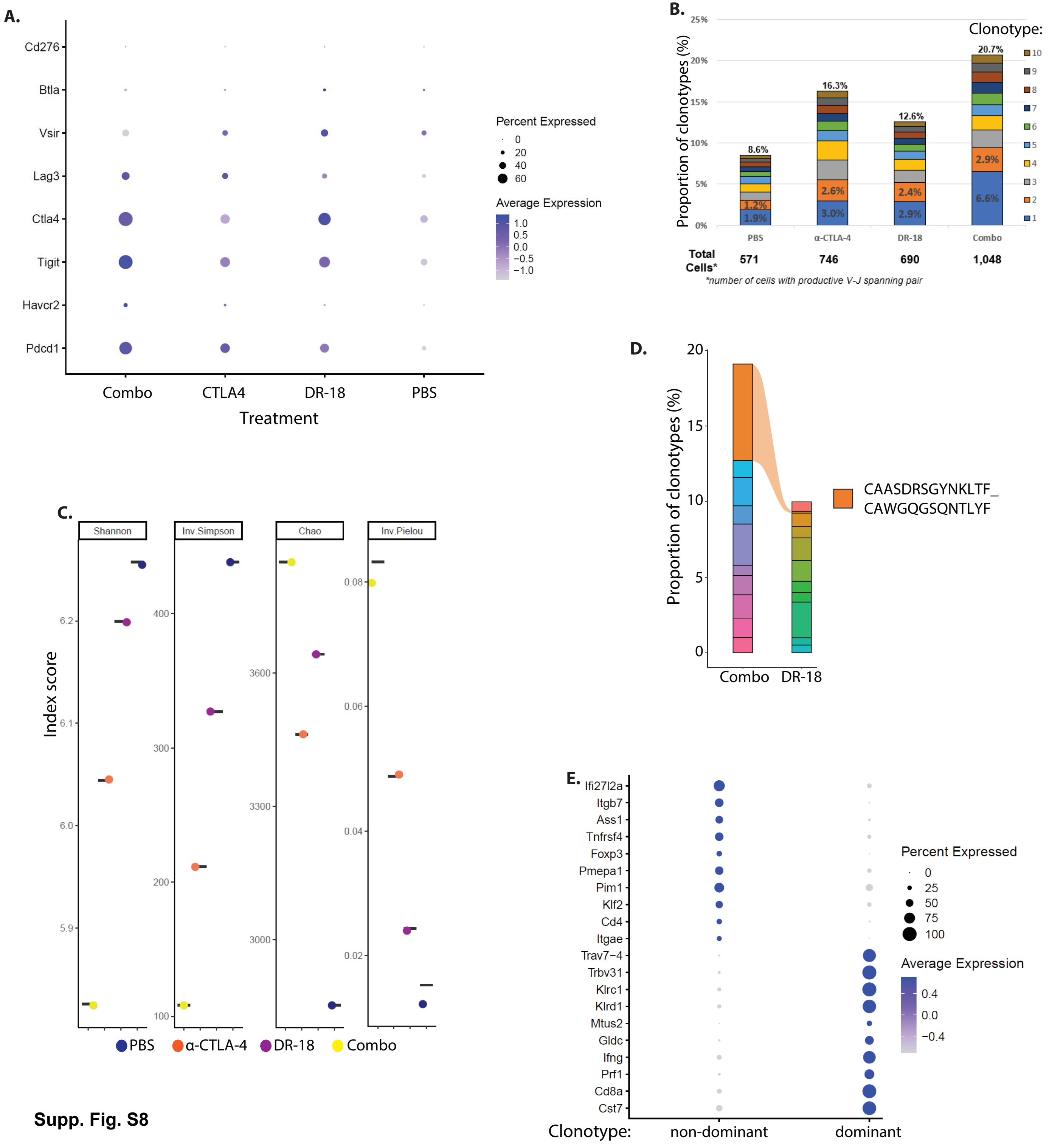
A.
Cd276
Btla
Vsir
Lag3
Ctla4
Tigit
Havcr2
Pdcd1
Combo
CTLA4
DR-18
PBS
Treatment
Percent Expressed
0
20
40
60
Average Expression
1.0
0.5
0.0
−0.5
−1.0
B.
Proportion of clonotypes (%)
Clonotype:
8.6%
16.3%
12.6%
20.7%
1.2%
1.9%
2.6%
3.0%
2.4%
2.9%
2.9%
6.6%
PBS
α-CTLA-4
DR-18
Combo
Total Cells*
571
746
690
1,048
*number of cells with productive V-J spanning pair
C.
Shannon
Inv.Simpson
Chao
Inv.Pielou
Index score
PBS
α-CTLA-4
DR-18
Combo
D.
Proportion of clonotypes (%)
Combo
DR-18
CAASDRSGYNKLTF_
CAWGQGSQNTLYF
E.
Ifi27l2a
Itgb7
Ass1
Tnfrsf4
Foxp3
Pmepa1
Pim1
Klf2
Cd4
Itgae
Trav7−4
Trbv31
Klrc1
Klrd1
Mtus2
Gldc
Ifng
Prf1
Cd8a
Cst7
Clonotype:
non-dominant
dominant
Percent Expressed
0
25
50
75
100
Average Expression
0.4
0.0
−0.4

Supp. Fig. S8

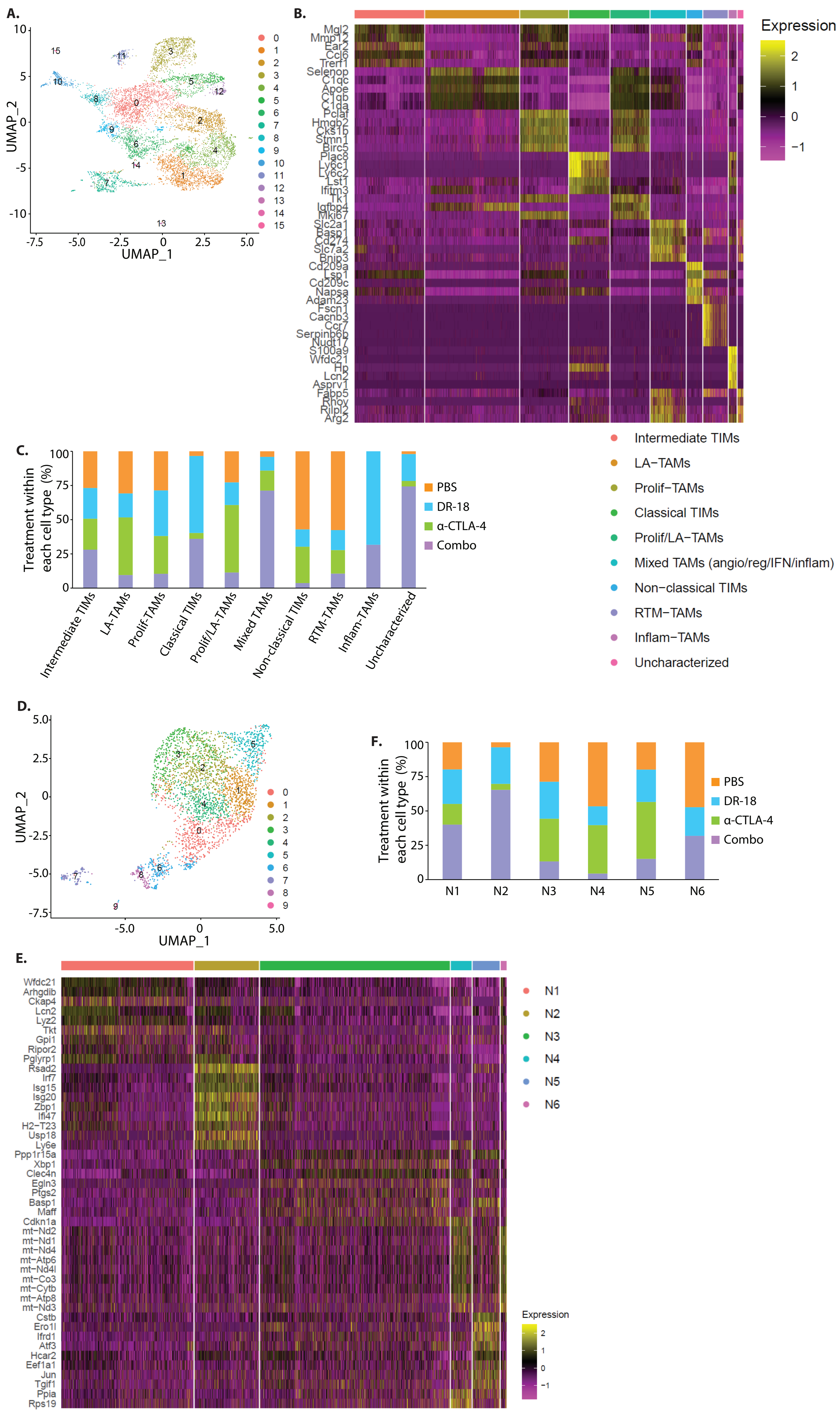
A.
UMAP_1
UMAP_2
0
1
2
3
4
5
6
7
8
9
10
11
12
13
14
15
B.
Expression
Mgl2
Mmp12
Ear2
Ccl6
Trerf1
Selenop
C1qc
Apoe
C1qb
C1qa
Pclaf
Hmgb2
Cks1b
Stmn1
Birc5
Plac8
Ly6c1
Ly6c2
Lst1
Ifitm3
Tk1
Igfbp4
Mki67
Slc2a1
Basp1
Cd274
Slc7a2
Bnip3
Cd209a
Lsp1
Cd209c
Napsa
Adam23
Fscn1
Cacnb3
Ccr7
Serpinb6b
Nudt17
S100a9
Wfdc21
Hp
Lcn2
Asprv1
Fabp5
Rhov
Rilpl2
Arg2
C.
Treatment within each cell type (%)
PBS
DR-18
α-CTLA-4
Combo
Intermediate TIMs
LA-TAMs
Prolif-TAMs
Classical TIMs
Prolif/LA-TAMs
Mixed TAMs
Non-classical TIMs
RTM-TAMs
Inflam-TAMs
Uncharacterized
Intermediate TIMs
LA−TAMs
Prolif−TAMs
Classical TIMs
Prolif/LA−TAMs
Mixed TAMs (angio/reg/IFN/inflam)
Non−classical TIMs
RTM−TAMs
Inflam−TAMs
Uncharacterized
D.
UMAP_1
UMAP_2
F.
Treatment within each cell type (%)
N1
N2
N3
N4
N5
N6
PBS
DR-18
α-CTLA-4
Combo
E.
Wfdc21
Arhgdib
Ckap4
Lcn2
Lyz2
Tkt
Gpi1
Ripor2
Pglyrp1
Rsad2
Irf7
Isg15
Isg20
Zbp1
Ifi47
H2−T23
Usp18
Ly6e
Ppp1r15a
Xbp1
Clec4n
Egln3
Ptgs2
Basp1
Maff
Cdkn1a
mt−Nd2
mt−Nd1
mt−Nd4
mt−Atp6
mt−Nd4l
mt−Co3
mt−Cytb
mt−Atp8
mt−Nd3
Cstb
Ero1l
Ifrd1
Atf3
Hcar2
Eef1a1
Jun
Tgif1
Ppia
Rps19
N1
N2
N3
N4
N5
N6
Expression

Supp. Fig. S9

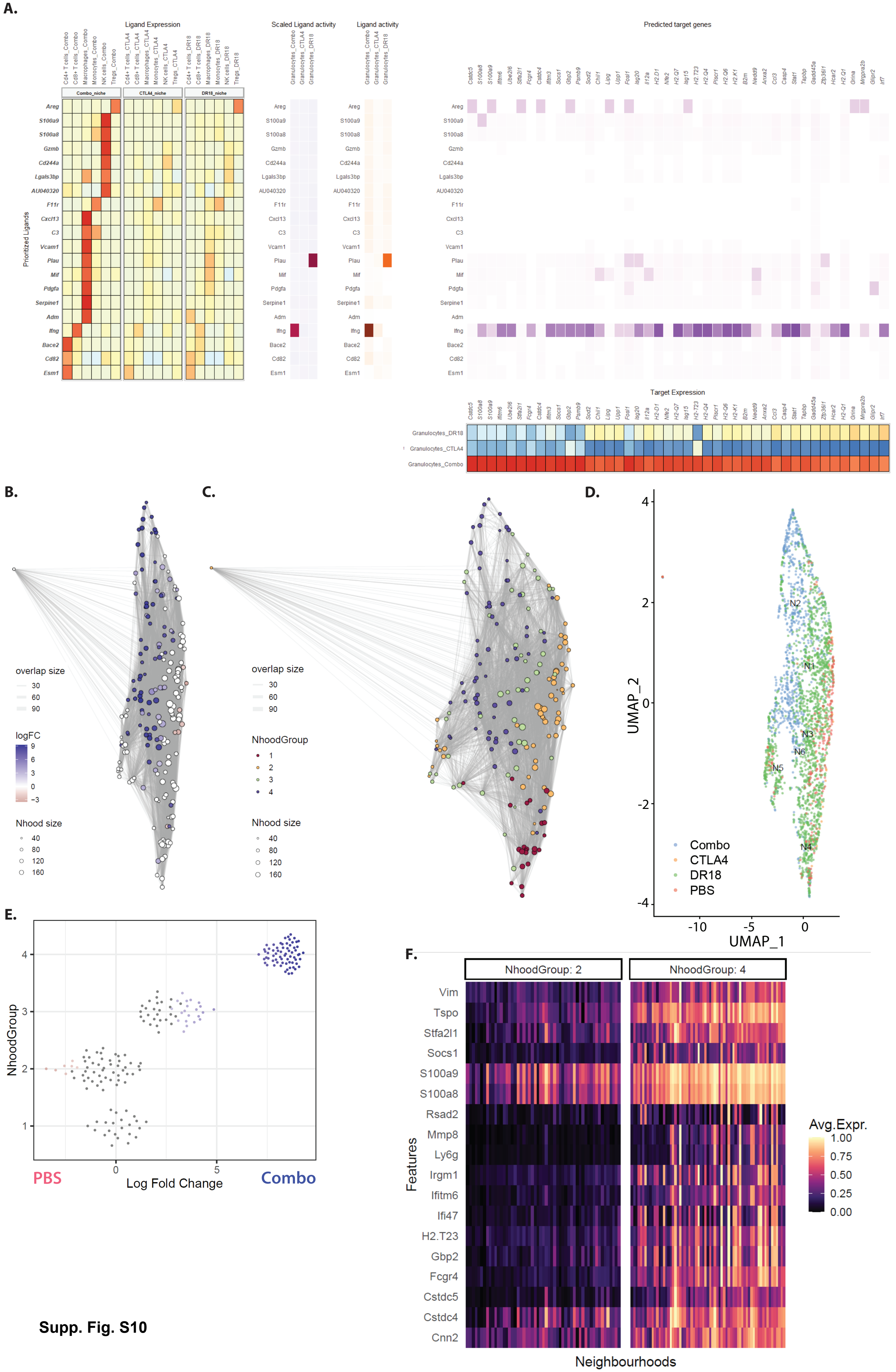
A.
Ligand Expression
Scaled Ligand activity
Ligand activity
Predicted target genes
Combo_niche
CTLA4_niche
DR18_niche
Prioritized Ligands
Areg
S100a9
S100a8
Gzmb
Cd244a
Lgals3bp
AU040320
F11r
Cxcl13
C3
Vcam1
Plau
Mif
Pdgfa
Serpine1
Adm
Ifng
Bace2
Cd82
Esm1
Target Expression
Granulocytes_DR18
Granulocytes_CTLA4
Granulocytes_Combo
B.
overlap size
30
60
90
logFC
9
6
3
0
−3
Nhood size
40
80
120
160
C.
overlap size
30
60
90
NhoodGroup
1
2
3
4
Nhood size
40
80
120
160
D.
UMAP_2
UMAP_1
N1
N2
N3
N4
N5
N6
Combo
CTLA4
DR18
PBS
E.
NhoodGroup
Log Fold Change
PBS
Combo
F.
NhoodGroup: 2
NhoodGroup: 4
Features
Vim
Tspo
Stfa2l1
Socs1
S100a9
S100a8
Rsad2
Mmp8
Ly6g
Irgm1
Ifitm6
Ifi47
H2.T23
Gbp2
Fcgr4
Cstdc5
Cstdc4
Cnn2
Avg.Expr.
1.00
0.75
0.50
0.25
0.00
Neighbourhoods

Supp. Fig. S10
